# Selective Fractionation and Valorization of Lithium Refinery Residues: A Critical Review

**DOI:** 10.3390/molecules31193539

**Published:** 2026-10-04

**Authors:** Zhizhao Song, Wenting Xu, Qingjun Guan, Juan Li, Honghu Tang, Fenghui Wu

**Affiliations:** 1 School of Resource Environment and Safety Engineering, Hunan University of Science and Technology, Xiangtan 411201, China; 2 Hunan Province Key Laboratory of Coal Resources Clean-Utilization and Mine Environment Protection, Xiangtan 411201, China; 3 School of Minerals Processing and Bioengineering, Central South University, Changsha 410083, China; 4 Faculty of Biological and Chemical Engineering, Panzhihua University, Panzhihua 617000, China

**Keywords:** lithium refinery residue, selective fractionation, rare metals, valorization, resource recovery

## Abstract

The rapid expansion of the lithium industry has generated large quantities of lithium refinery residue (LRR), whose stockpiling and landfilling cause environmental risks and the loss of potentially recoverable resources. This review critically examines LRR generated from the processing of spodumene and lepidolite, with particular emphasis on how differences in their mineralogical and chemical characteristics affect subsequent utilization and resource-recovery strategies. Current utilization pathways are classified into bulk utilization and selective fractionation followed by product-oriented valorization. Bulk utilization mainly includes direct incorporation into construction materials, activated cementitious materials, and sintered products, offering high residue-consumption capacity but generally limited added value. Selective fractionation employs leaching, flotation, magnetic separation, and gravity separation to separate Si-Al, Ca-S, and Fe-bearing fractions and to recover residual valuable elements, including Li, Ta, Nb, Rb, and Cs. The removal or stabilization of hazardous constituents, particularly F, Be, and Tl, is also evaluated as a prerequisite for safe valorization. The recovered fractions can subsequently be converted into products such as aluminosilicate powders, porous ceramics, zeolites, glass-fiber feedstocks, high-strength α-hemihydrate gypsum, and anhydrite II. Finally, the reviewed pathways are critically compared in terms of technical feasibility, resource efficiency, environmental risks, economic potential, technological maturity, and scale-up constraints. Priority is given to integrated processing strategies that combine hazardous-element control, selective recovery of valuable components, and large-volume utilization of bulk fractions to improve the overall sustainability and industrial viability of LRR valorization.

## 1. Introduction

With the rapid development of the lithium battery industry, the demand for lithium salt products—such as lithium carbonate and lithium hydroxide—continues to grow [1,2], and the scale of lithium mining operations is steadily expanding. Spodumene and lepidolite are major raw materials for lithium salt production in China, and their thermal and hydrometallurgical processing generates substantial quantities of solid residues, herein referred to as lithium refinery residue (LRR). These residues are classified as spodumene-derived LRR and lepidolite-derived LRR, respectively. The quantity of LRR generated varies considerably depending on the ore type, raw material grade, and extraction process. Existing studies indicate that producing 1 t of Li_2_CO_3_ from spodumene generates approximately 8–10 t of LRR [3], whereas lepidolite processing produces substantially more residue because of its lower lithium grade and higher gangue content, typically about 20–30 t of LRR per tonne of Li_2_CO_3_ [4]. In Yichun, China, approximately 4.4 million tonnes of lithium slag were generated in 2022, with annual generation projected to reach approximately 15 million tonnes under the 2025 production scenario [5,6]. Outside China, lithium refinery plants in Australia have been projected to generate approximately 1.4 million tonnes of LRR annually. Currently, the comprehensive utilization rate of LRR remains below 30%, with most residues still stockpiled or landfilled. This not only results in the loss of potentially recoverable resources but also creates environmental risks because harmful elements such as F, Tl, and Be may migrate into soil and water through leaching [7,8,9]. Therefore, developing efficient, cost-effective, and environmentally safe technologies for the valorization of LRR—thereby enabling the recovery of valuable components and industrial application—is essential to the sustainable development of the lithium salt industry.

Extensive research has been conducted on the valorization of LRR, covering applications such as cement admixtures, concrete additives, geopolymers, and ceramics [10,11,12]. However, current practices primarily focus on low-value construction material applications, resulting in limited product added value. From a resource perspective, LRR is a secondary resource containing valuable components that can be economically recovered [13]. In addition to major constituents like silicon (Si), aluminum (Al), calcium (Ca), and sulfur (S), some LRR samples contain residual rare metals—such as lithium (Li), rubidium (Rb), cesium (Cs), tantalum (Ta), and niobium (Nb)—which can be recovered through selective fractionation methods such as leaching, magnetic separation, and gravity separation [14,15,16]. However, the presence of harmful elements and substances—such as soluble salts, Be, Tl, and F—can compromise the quality of high-value products and affect their long-term stability [17]. Therefore, prior to high-value utilization, measures for selective removal or stabilization should be implemented, tailored to the speciation and host phases of the harmful elements and the intended application of the product. Existing reviews largely emphasize low-value disposal, offering limited systematic analysis of separation and recovery technologies for valuable components and lacking an integrated perspective on detoxification and bulk utilization.

Relevant studies were selected and organized according to four themes: the physicochemical characteristics of LRR, bulk utilization, component-specific separation and recovery, and product-oriented valorization of the separated components. Building on the physicochemical differences between spodumene-derived and lepidolite-derived LRR, this review evaluates two main valorization strategies: bulk utilization and selective fractionation followed by product-oriented valorization. It further examines how the occurrence and separability of Si-Al, Ca-S, Fe-bearing, and residual valuable-metal components influence the selection of separation methods and downstream products. By comparing the technical performance, environmental implications, economic considerations, and industrial applicability of these pathways, this review identifies key challenges and research needs for integrated LRR processing and scale-up.

## 2. Sources and Physicochemical Properties of LRR

### 2.1. Sources and Classification of LRR

Primary lithium resources can be broadly divided into brines and lithium-bearing minerals [18]. Among ore-based resources, spodumene and lepidolite are the primary raw materials for lithium salt production [5,14,19,20,21,22,23,24]. Spodumene is a Li-Al-Si-bearing mineral, whereas lepidolite additionally contains relatively high contents of K and F and may also host Rb and Cs. These compositional differences in the primary feedstocks, together with the reagents introduced during lithium extraction, largely determine the chemical and mineralogical characteristics of the corresponding LRR. The resulting compositions of spodumene-derived and lepidolite-derived LRR are summarized in Table 1. Accordingly, the solid LRR considered in this review are primarily classified as spodumene-derived and lepidolite-derived LRR, while other primary lithium resources and their associated waste streams are briefly discussed in Section 2.1.3.

#### 2.1.1. Sources of Spodumene-Derived LRR

**Figure 1:**
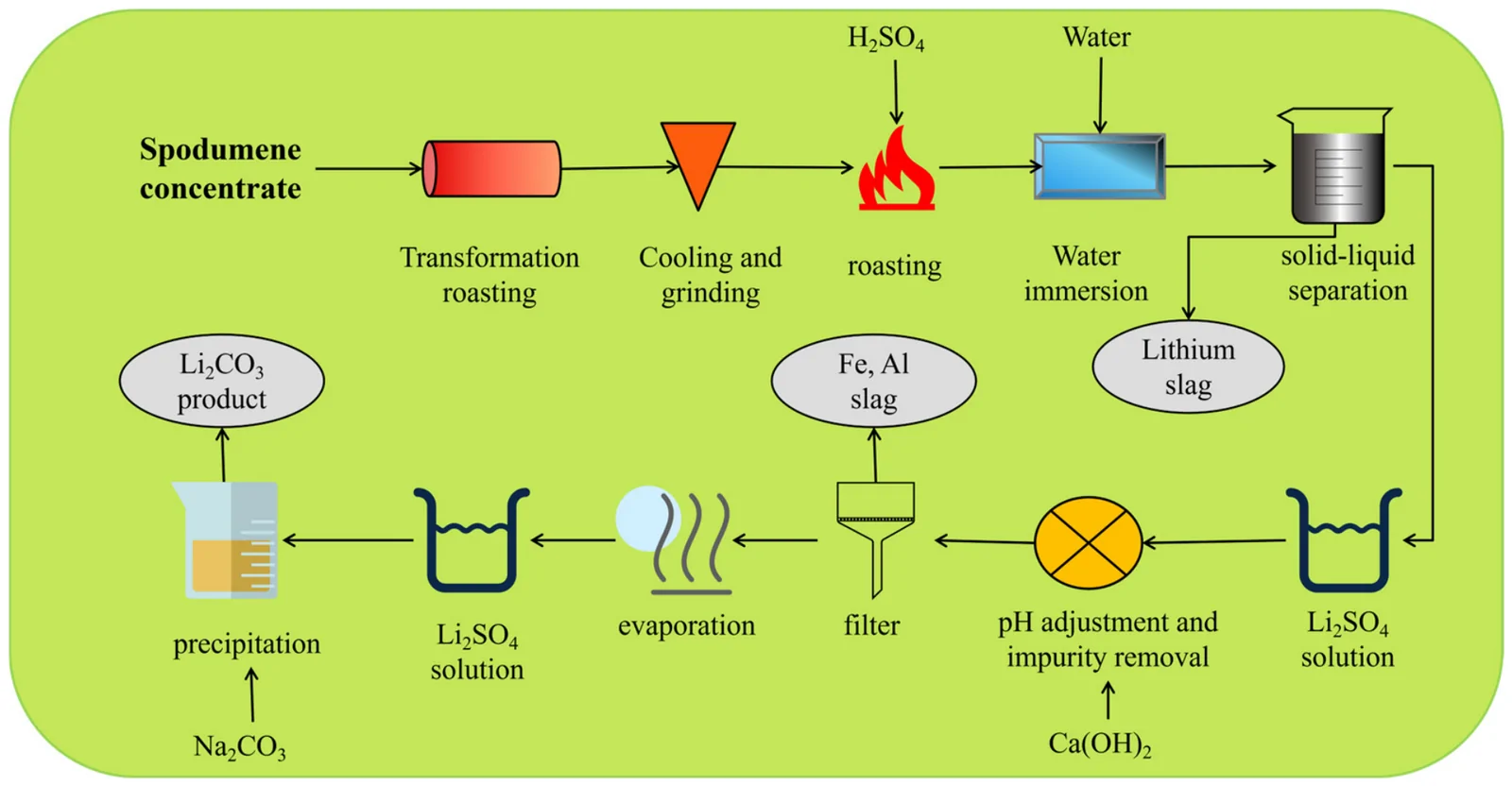
Process flow diagram for lithium extraction from spodumene via the sulfuric acid method.

Currently, the industrial extraction of lithium from spodumene primarily employs the sulfuric acid method. This process generally comprises four steps: high-temperature phase transformation, sulfuric acid roasting, water leaching and impurity removal, and lithium precipitation [25] (Figure 1). Natural α-spodumene possesses a dense structure and low reactivity. Direct sulfuric acid leaching struggles to achieve the efficient release of lithium. Therefore, a high-temperature phase transformation at 1000–1100 °C is required to convert the material into β-spodumene, which possesses a porous structure and high chemical reactivity. Subsequently, the material is cooled and pulverized, mixed with concentrated sulfuric acid, and subjected to acid roasting at approximately 200–300 °C. This process replaces Li^+^ ions in the crystal lattice with H^+^ ions, converting the lithium into water-soluble Li_2_SO_4_. The roasted product undergoes water leaching to yield a lithium-bearing leachate and LRR. Fe and Al impurities are then removed by adjusting the pH of the leachate. Finally, following evaporation and concentration, Na_2_CO_3_ is added to precipitate Li_2_CO_3_ [26,27,28].

Spodumene processing can also employ techniques such as sulfate roasting, chlorination roasting, and alkaline pressure leaching. Sulfate roasting utilizes media like Na_2_SO_4_ and K_2_SO_4_. Compared with concentrated sulfuric acid, it offers advantages such as reduced corrosiveness and lower acidic exhaust emissions, though it faces challenges including high reaction temperatures, significant salt consumption, and the co-leaching of impurities. Chlorination roasting offers high reaction efficiency, but the potential generation of corrosive, environmentally hazardous chlorine-containing gases—combined with the high cost of necessary corrosion-resistant equipment—limits its practical application [27,28]. Alkaline pressure leaching utilizes reagents such as Na_2_CO_3_ and NaOH to decompose spodumene under high-temperature and high-pressure conditions, yielding high-purity lithium carbonate. However, it imposes stringent requirements on equipment and process control, and entails high investment and operating costs [22]. In contrast, although the sulfuric acid method suffers from drawbacks such as high energy consumption during roasting, equipment corrosion, and the generation of large quantities of sulfate-containing LRR, it remains the primary process for producing lithium carbonate from spodumene due to its high lithium recovery rate, stable product quality, mature technology, and established industrial application [19,26,28,29,30].

#### 2.1.2. Sources of Lepidolite-Derived LRR

**Figure 2:**
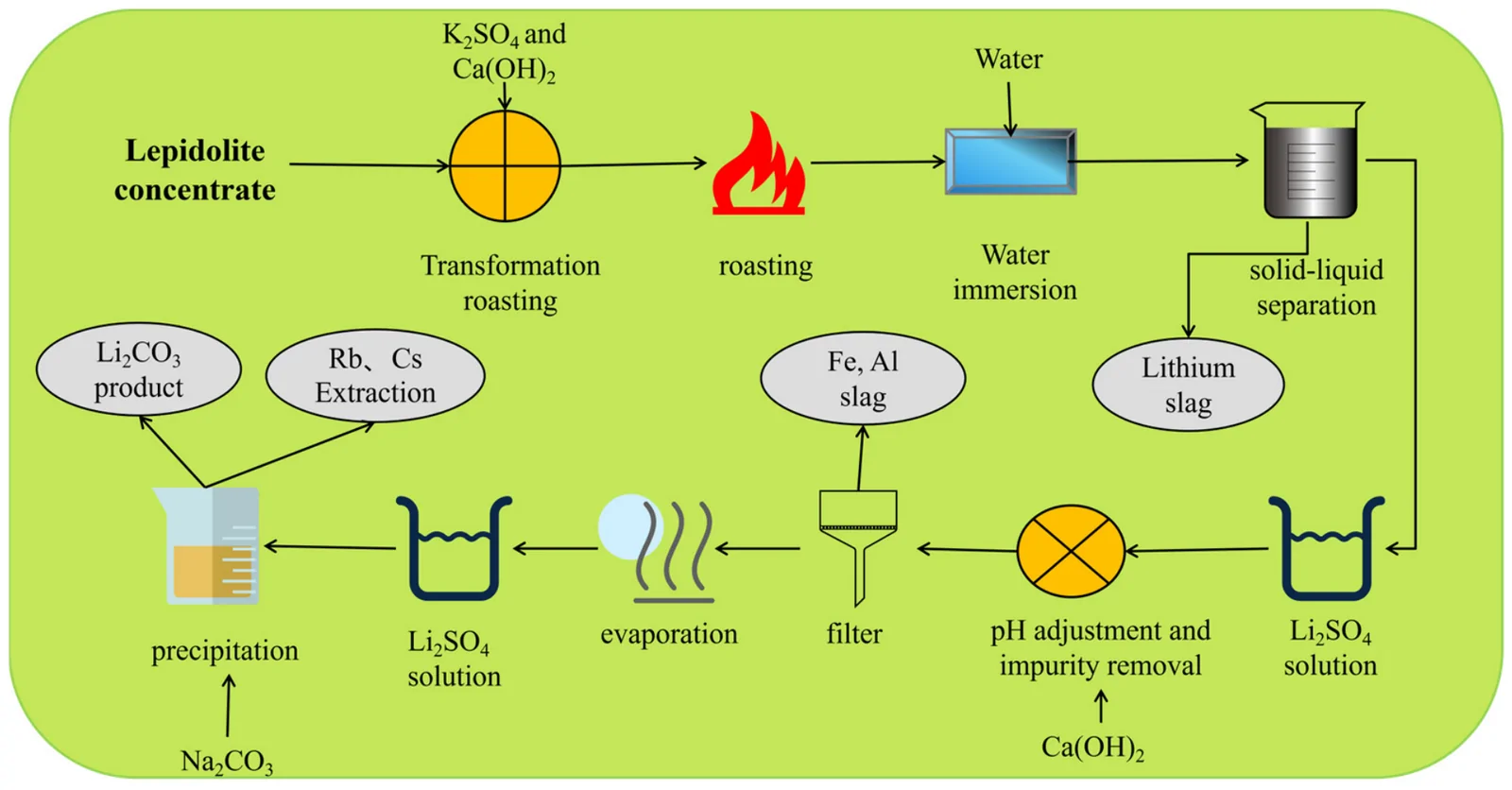
Process flow diagram for lithium extraction from lepidolite via sulfate roasting.

A common method for extracting lithium from lepidolite is a combined sulfate roasting process with calcium hydroxide [25] (Figure 2). In this process, lepidolite concentrate is roasted with K_2_SO_4_ and Ca(OH)_2_, inducing ion exchange and mineral phase reconstruction within the crystal lattice, thereby converting Li, Rb, and Cs into water-soluble sulfates [31,32]. Simultaneously, the CaO generated from the thermal decomposition of Ca(OH)_2_ promotes mineral decomposition and facilitates the formation of insoluble F-bearing phases, such as CaF_2_. Following water leaching of the roasted product, Li, Rb, and Cs enter the leachate, while residues—including quartz, calcium-aluminum silicates, and F-bearing minerals—form the lepidolite-derived LRR [31,33]. The leachate undergoes pH adjustment to remove Fe and Al impurities, followed by concentration and precipitation to produce Li_2_CO_3_ [34,35].

Compared with acid, alkaline, and chlorination roasting methods, the sulfate roasting process offers advantages such as higher lithium leaching rates, a simple water-leaching procedure, reduced equipment corrosion, and the ability to immobilize fluoride as CaF_2_. Consequently, it has become one of the commonly adopted processes for extracting lithium from lepidolite [36].

#### 2.1.3. Other Primary Lithium Resources and Associated Residues

In addition to spodumene and lepidolite, lithium is recovered from other hard-rock resources, including petalite and lithium-bearing mica minerals such as zinnwaldite, as well as from brines [18,23]. Lithium extraction from hard-rock minerals generally involves thermal and hydrometallurgical treatment, whereas brine processing mainly relies on evaporation/precipitation, adsorption, ion exchange, and other separation processes. These different processing routes generate distinct waste streams: hard-rock processing mainly produces solid mineral and leaching residues, whereas brine-based processes generate saline liquid streams, precipitated salts, and impurity-removal solids. Although solid residues from other hard-rock lithium resources may also have potential for selective fractionation and valorization, studies on their composition, phase occurrence, separability, and downstream utilization remain relatively limited. Therefore, the subsequent detailed discussion focuses primarily on spodumene- and lepidolite-derived LRR, for which residue characterization and valorization studies are more extensive.

### 2.2. Physicochemical Properties of LRR

#### 2.2.1. Physical Properties of LRR

**Figure 3:**
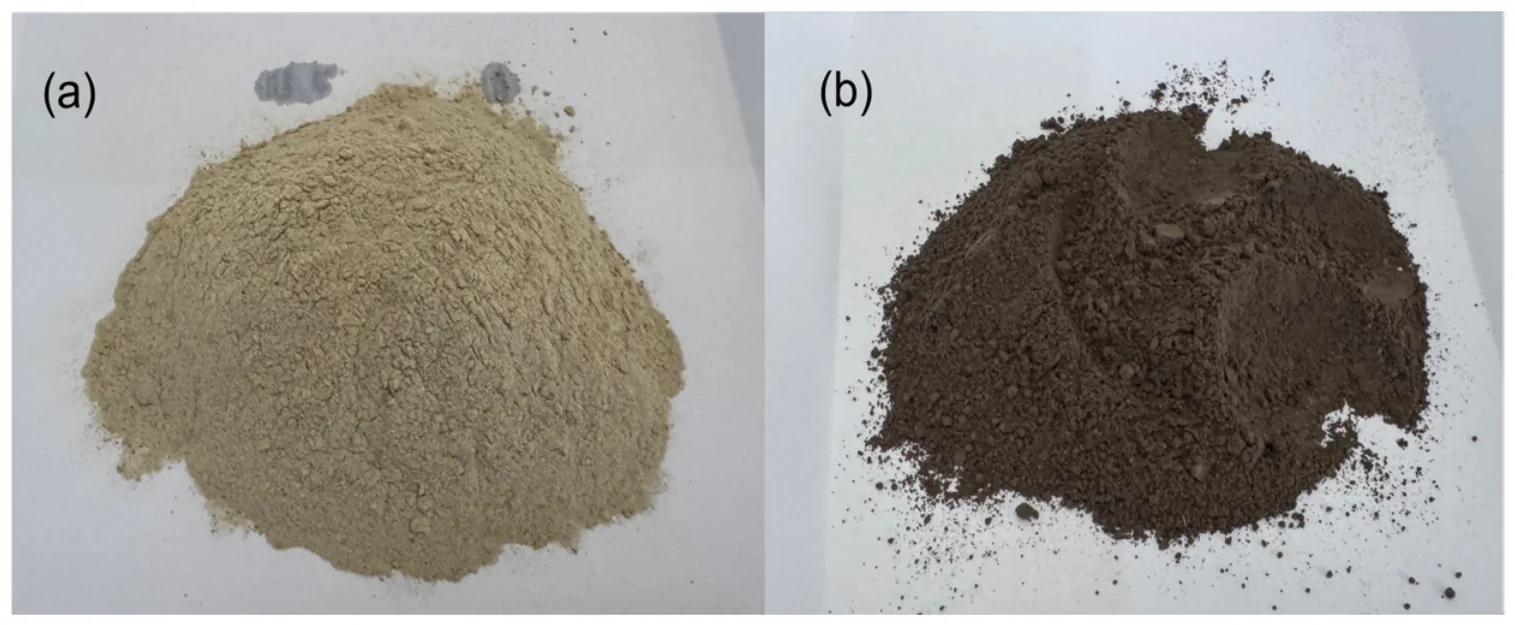
Visual characteristics of (**a**) spodumene-derived LRR and (**b**) lepidolite-derived LRR.

**Figure 4:**
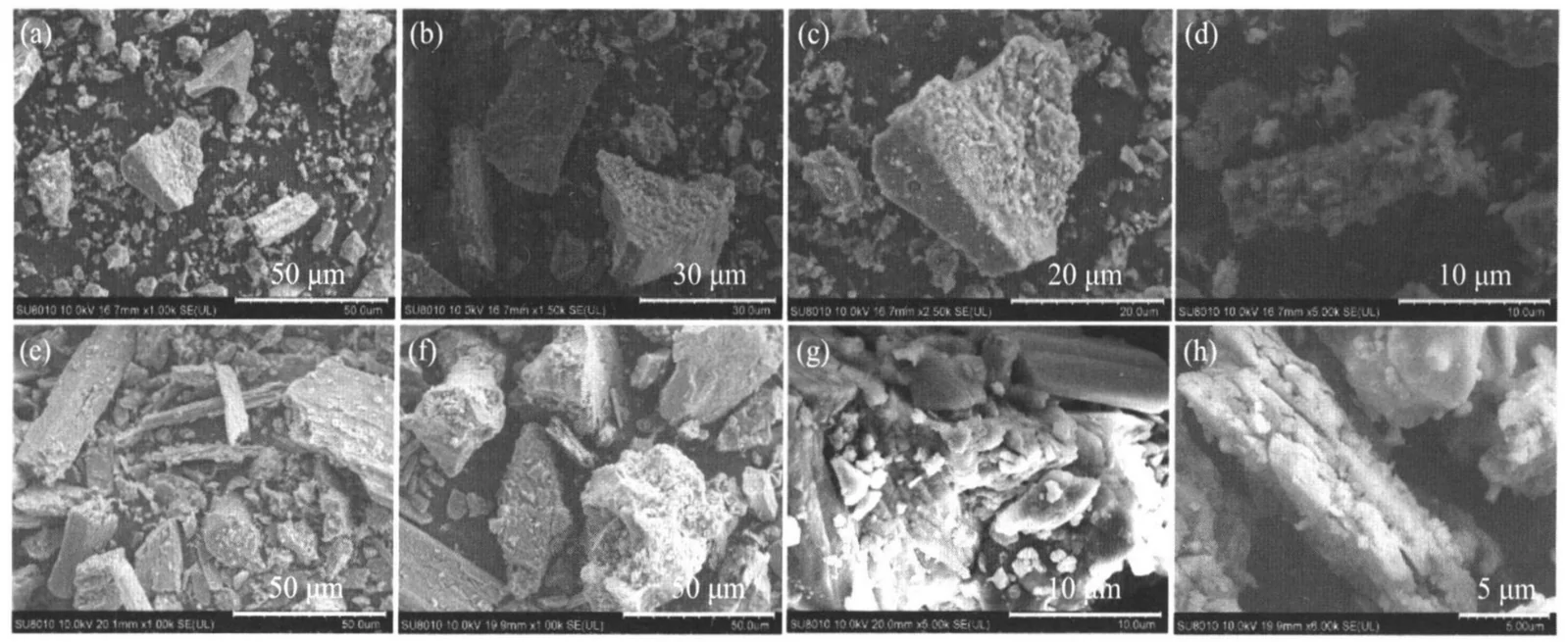
SEM images of lepidolite-derived LRR (**a**–**d**) and spodumene-derived LRR (**e**–**h**) [38].

As shown in Figure 3, the two types of LRR show a marked color contrast: spodumene-derived LRR is yellow, whereas lepidolite-derived LRR is black [7]. This color difference is primarily attributed to chromogenic elements, such as manganese, in lepidolite. Residual manganese in lepidolite-derived LRR darkens the residue, whereas the relatively low content of such elements in spodumene-derived LRR results in a lighter color [37]. As illustrated in Figure 4, both residues consist of irregularly shaped particles [38]. Both types of LRR have densities ranging from 2.5 to 3.5 g/cm^3^, feature porous structures with rough surfaces, and generally have particle sizes in the 1–100 μm range [39,40].

#### 2.2.2. Chemical Properties of LRR

**Figure 5:**
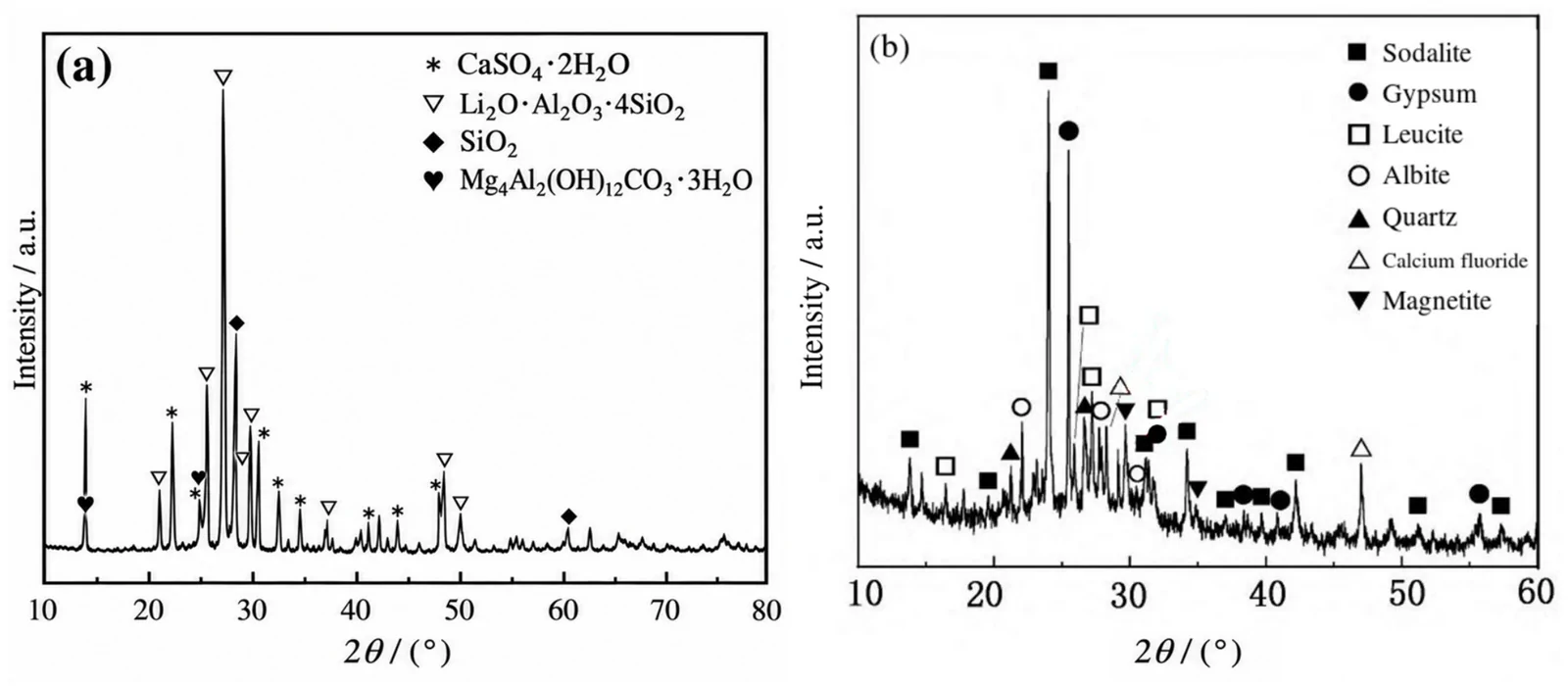
XRD patterns of (**a**) spodumene-derived LRR [41] and (**b**) lepidolite-derived LRR [42].

**Figure 6:**
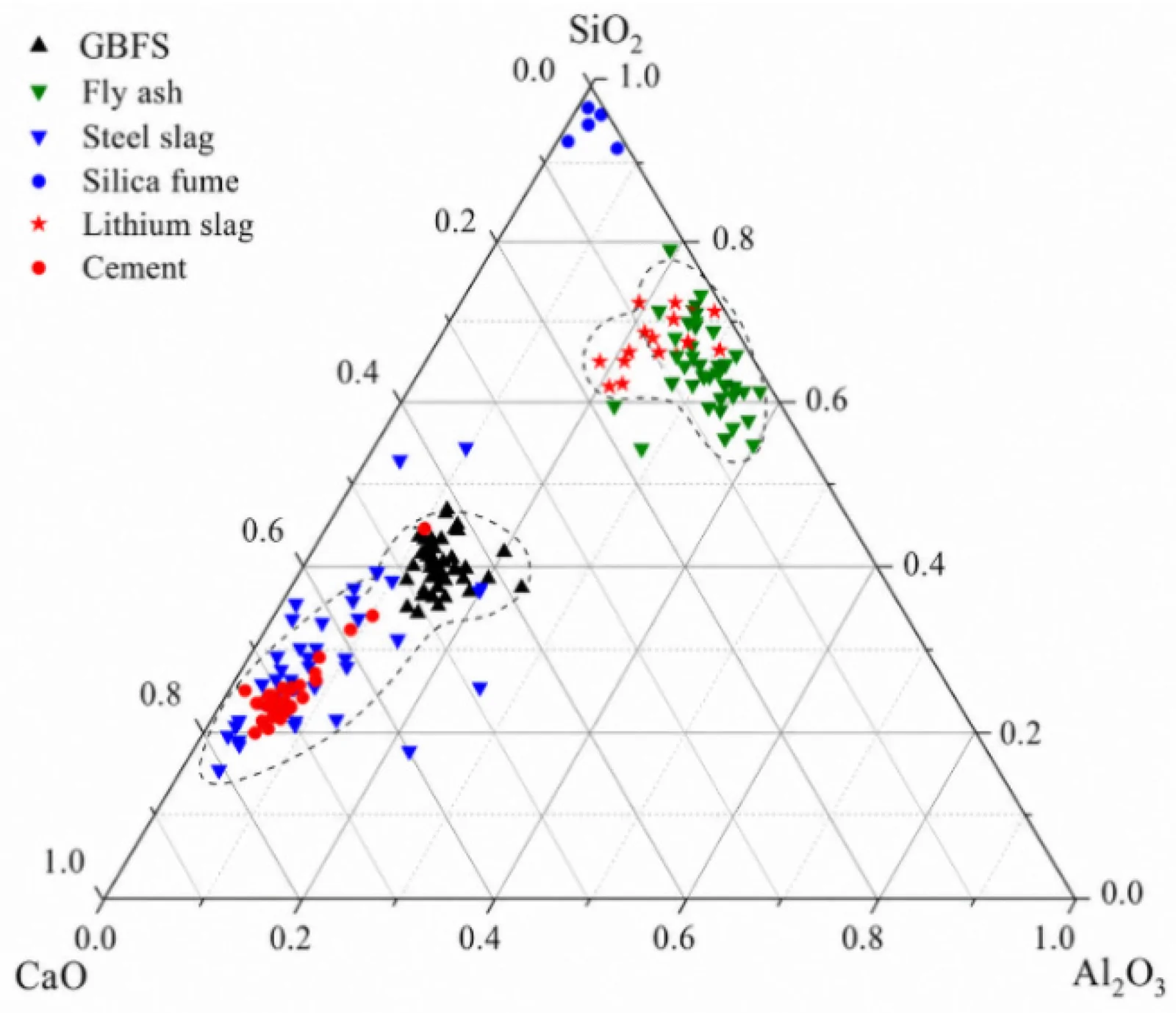
Comparison of the oxide compositions of LRR and other supplementary cementitious materials [45].

The two LRR types differ markedly in chemical composition. As summarized in Table 1, their major constituents include SiO_2_ and Al_2_O_3_, alongside CaO, SO_3_, Na_2_O, K_2_O, MgO, Fe_2_O_3_, and F, as well as minor amounts of residual Li, Rb, Cs, Ta, and Nb. Owing to treatments such as salt roasting, salt-based pressure leaching, or sulfate roasting, lepidolite-derived LRR exhibits higher contents of Na_2_O, K_2_O, and F. Figure 5 presents the XRD patterns for spodumene-derived and lepidolite-derived LRR [41,42]. The patterns reveal that the primary mineral phases in both materials are aluminosilicates and gypsum, a finding consistent with their chemical compositions. Compared to spodumene-derived LRR, lepidolite-derived LRR exhibits a more complex composition. In addition to aluminosilicates and gypsum, it contains mineral phases such as calcium fluoride, quartz, and magnetite. Figure 6 shows that the chemical composition of LRR is similar to that of fly ash, indicating weak self-hardening properties and latent pozzolanic activity [42,43,44,45].

In addition to major chemical constituents, LRR may contain hazardous elements such as F, Be, Tl, As, Cd, Pb, Cr, and Mn [17,46]. These elements primarily originate from associated components in the raw ore and may remain in the solid phase following roasting and leaching processes. Heavy metal contents in spodumene-derived LRR are generally low. For instance, Fe and Mn levels are typically below 0.5%, and Pb and Cd are undetectable in some samples, indicating that the primary environmental risk stems from residual sulfates [19]. In contrast, lepidolite-derived LRR exhibits higher levels of F, Be, and Tl. Typical samples contain approximately 2.80% F, with Be and Tl concentrations of 19.97 and 2.74 μg/g, respectively [47], while samples from different enterprises show Be and Tl ranges of 21–78 mg/kg and 4–21 mg/kg, respectively [17]. Acidic conditions can promote the leaching of Be and F-bearing mineral phases, whereas reducing conditions may enhance Tl mobility, posing risks to soil, groundwater, and ecosystems [17,48]. According to GB 5085.3-2007, the regulatory limits for total Be and inorganic fluorides in leachate are 0.02 mg/L and 100 mg/L, respectively [49]. GB 3838-2002 specifies reference limits of 1.0 mg/L for F, 0.002 mg/L for Be, and 0.0001 mg/L for Tl in surface water [50]. Therefore, prior to the valorization of LRR, environmental risk assessments—integrating both total hazardous element content and leaching characteristics—should be conducted, and components exceeding limits must be removed or stabilized.

Overall, these physicochemical characteristics govern the downstream utilization of LRR. The Si-Al-rich composition and latent pozzolanic activity provide a basis for cementitious utilization, while the Si-Al-rich mineral framework and alkali-bearing constituents provide favorable compositional characteristics for sintered products. Conversely, Ca-S and Fe-bearing phases, hazardous constituents, and residual valuable elements create a need for selective separation and recovery. These composition–property relationships therefore underpin the two utilization strategies discussed in Section 3 and Section 4.

**Table 1:** Chemical compositions of LRR from different sources (wt%).

Types of LRR	Origin	SiO_2_	Al_2_O_3_	CaO	SO_3_	K_2_O	Na_2_O	MgO	Fe_2_O_3_	F	LOI
**Spodumene-derived LRR**	Jiangxi [51]	49.34	20.46	16.60	10.18	-	-	0.54	1.31	-	-
	Jiangxi [52]	57.22	21.28	8.41	-	1.63	0.28	0.15	0.87	-	5.83
	Hubei [53]	51.20	20.60	6.40	7.20	-	-	1.20	0.80	-	-
	Shandong [54]	56.26	17.39	9.83	-	1.21	1.32	0.26	0.96	-	5.65
	Sichuan [55]	53.22	17.11	10.11	7.15	0.53	0.33	0.41	1.48	-	8.25
	Sichuan [56]	41.96	16.49	4.15	5.20	0.66	0.08	0.23	1.04	0.21	27.53
**Lepidolite-derived LRR**	Jiangxi [4]	47.62	21.56	2.02	0.03	3.05	10.68	0.12	0.48	-	0.14
	Jiangxi [11]	50.25	22.75	2.13	0.03	3.22	11.27	0.13	0.51	-	0.15
	Jiangxi [56]	24.32	11.21	16.24	22.12	4.33	5.38	0.43	2.26	3.10	8.77
	Jiangxi [57]	42.30	18.70	12.40	5.60	1.40	2.10	1.80	3.20	2.56	4.20
	Sichuan [58]	52.21	20.60	4.63	9.18	0.26	-	0.16	0.84	-	11.39

Note: “-” indicates not reported in the original text.

## 3. Bulk Utilization of Whole LRR

LRR valorization can be broadly divided into bulk utilization of the whole residue and selective fractionation followed by component-oriented valorization. This section focuses on the former strategy and covers direct incorporation without activation, activated cementitious utilization, and the manufacture of sintered products. These methods offer advantages such as high consumption capacity and the ability to immobilize certain hazardous elements [57,59]. However, challenges remain regarding high activation costs, significant energy consumption during sintering, and the need to verify long-term stability [60]. Furthermore, valuable components are not effectively recovered, resulting in low product added value [13].

### 3.1. Direct Incorporation Without Activation

Direct incorporation refers to the practice of adding LRR—subjected only to crushing and screening rather than specialized activation or purification—into engineering materials, where it primarily functions through filler effects, aggregate-skeleton formation, and limited pozzolanic reactions. Wu et al. [61] utilized LRR to improve silty clay and validated the results through a field test section. Xue et al. [62] found that increasing the LRR content in backfill slurry to 30% reduced the structural recovery ratio to 0.639, indicating that excessive incorporation hinders structural recovery after pumping stops. Zhang et al. [63] produced high-solid-waste-content concrete by partially replacing natural sand with LRR, achieving a freeze–thaw resistance grade of F150–F200. While this method involves simple pretreatment and relatively low costs, the optimal dosage varies depending on the specific application. While direct incorporation involves simple pretreatment and relatively low costs, the appropriate LRR dosage depends on the specific application. More importantly, direct incorporation does not necessarily ensure the long-term immobilization of hazardous constituents in LRR. Prolonged water exposure and changes in environmental pH may affect the mobility and leaching behavior of elements such as F, Be, and Tl, particularly in lepidolite-derived LRR [17,48]. Therefore, the long-term leaching stability of directly incorporated LRR should be evaluated under representative service conditions.

### 3.2. Preparation of Activated Cementitious Materials

Compared with direct incorporation, activation-based utilization aims to further exploit the latent reactivity of LRR by enhancing the availability of reactive Si-Al species. As shown in Figure 6, LRR exhibits weak self-hardening properties and latent pozzolanic activity. However, the high degree of polymerization of the aluminosilicate structure within the slag hinders the dissolution of Si-Al components, thereby limiting the formation of hydration gels and the development of cementing activity [64]. Consequently, appropriate activation methods are typically required to disrupt the mineral structure and promote the dissolution of Si-Al components, thereby enhancing the utilization efficiency of LRR in cementitious materials.

#### 3.2.1. Activation Treatment

**Figure 7:**
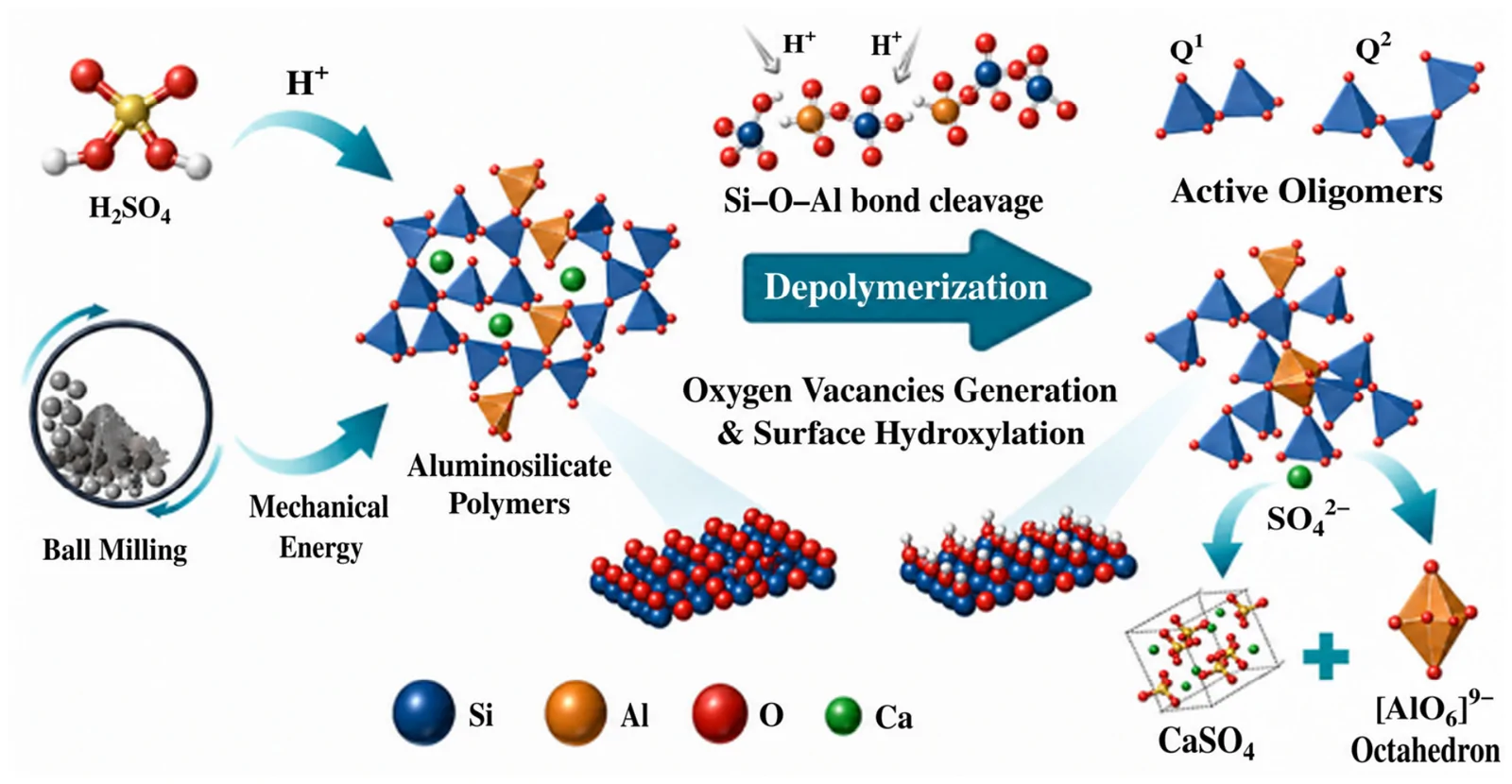
Schematic of sulfuric-acid-assisted mechanical activation of LRR [64].

Activation methods for LRR primarily fall into four categories: mechanical, thermal, chemical, and synergistic activation using other solid wastes. Mechanical activation refines particles through grinding, thereby increasing the reaction interface and disrupting the crystal structure. Yang et al. [65] achieved a 28-day activity index of 119.03% by grinding LRR to a particle size of approximately 2 μm. However, excessive grinding can lead to particle agglomeration, and Zha et al. [66] found that optimal results were obtained with a maximum particle size of 45 μm and a grinding time of about 15 min. Thermal activation increases the amorphous phase content through calcination. The optimal temperature varies by system but typically ranges from 550 to 700 °C [51,65]. While this method effectively enhances the reactivity of LRR, high-temperature calcination entails increased energy consumption. Chemical activation employs activators such as acids or alkalis to promote the dissolution of Si-Al components. Using combined sulfuric-acid treatment and mechanical grinding, Li et al. [64] prepared a binder containing 45% LRR to achieve a 28-day compressive strength of 86.75 MPa—surpassing the requirements for Grade 52.5 cement—with the reaction mechanism illustrated in Figure 7. However, there is an optimal dosage for activators, and excessive amounts may inhibit hydration [67]. Synergistic activation with other solid wastes utilizes alkaline materials—such as desulfurization ash, carbide slag, and magnesium slag—to provide an alkaline environment and reactive calcium components, thereby promoting the dissolution of silicon and aluminum from the LRR and the formation of hydration gels [59,66,68]. Road base materials prepared by Cao et al. [59] using magnesium slag to activate LRR met the strength specifications for Class I highways.

Overall, mechanical activation is operationally simple but electricity-intensive. Thermal activation effectively increases reactivity but requires significant energy. Chemical activation is highly effective but increases reagent costs. And synergistic activation of solid wastes enables the combined utilization of multiple waste streams, though the compatibility of raw materials and the stability of performance still require verification.

#### 3.2.2. Utilization in Cementitious Materials

The utilization of LRR in cement systems encompasses applications such as clinker raw meal components, active supplementary cementitious materials, and clinker-free binders [4,53,68,69]. Given the high sulfate content of LRR, the proportion of high-sulfur constituents must be carefully controlled. Moderate sulfate levels facilitate ettringite formation and pore filling, whereas excessive sulfate induces expansion and cracking [4]. Gao et al. [69] balanced sulfate and reactive alumina levels by adjusting the ratio of slag to desulfurization gypsum, at a 40 wt% replacement level, the 28-day strength exceeded that of pure cement by 12.7%, while both costs and carbon emissions were reduced. Miao et al. [53] leveraged the high sulfate content as an activator to produce supersulfated cement that is virtually clinker-free. Additionally, carbonation curing can compensate for the loss of early-stage strength associated with high replacement levels [70]. Although cementitious applications provide the largest potential outlet and are compatible with existing cement-production infrastructure, replacement levels remain relatively low due to limitations imposed by current standards.

In contrast, alkali-activated systems do not require clinker. LRR is a suitable precursor for geopolymer production because SiO_2_ and Al_2_O_3_ are its dominant constituents and its aluminosilicate phases exhibit latent pozzolanic reactivity. Under alkaline activation, reactive Si- and Al-bearing species are released and subsequently participate in the formation of the binding network. Therefore, the high abundance of Si–Al components and their activation potential provide the compositional basis for the utilization of LRR in geopolymer systems. A geopolymer prepared by Luo et al. [11] via a one-part mixing method achieved a 28-day compressive strength of 32.5 MPa at an LRR replacement level of 50%. When LRR was blended with ground granulated blast-furnace slag, the optimal LRR content was approximately 30%, resulting in compressive strengths of 51–71 MPa [71,72,73]. While these systems offer high replacement levels and strength, they face challenges regarding high activator costs and a lack of durability data [74]. Zhang et al. [60] produced uncalcined lightweight aggregates using a 4:1 blend of spodumene-derived LRR and lepidolite-derived LRR, achieving a total slag content exceeding 85%. They further employed CO_2_ curing to sequester approximately 830 kg of CO_2_ per tonne of material [75]. The primary advantage of these approaches is their capacity to consume large quantities of LRR, although they remain constrained by the economically viable transport radius.

**Figure 8:**
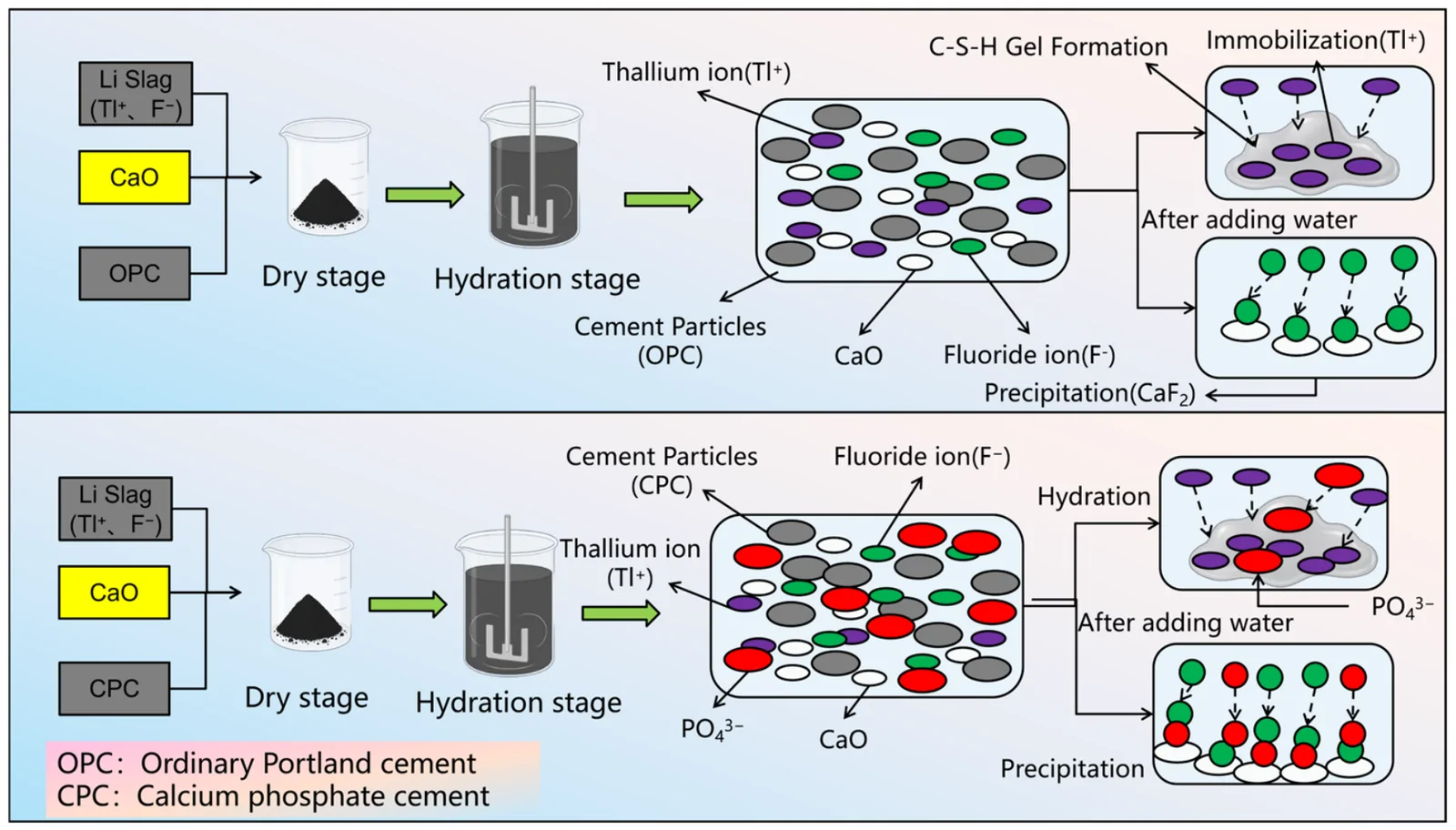
Schematic diagram of the synergistic immobilization mechanisms of Tl^+^ and F^−^ in LRR by two cement-based systems [57].

LRR-based cementitious materials can immobilize hazardous elements through mechanisms such as chemical precipitation, surface adsorption, physical encapsulation, ion substitution, and chemical bonding. Alhijjawi et al. [57] utilized a low-dosage cement–calcium oxide system to immobilize fluoride ions as CaF_2_ and immobilize Tl within C–S–H gel. This reduced the leaching concentration of Tl from 89 μg/L to 0.17 μg/L and decreased the F leaching concentration by approximately 74% (the mechanism is illustrated in Figure 8). Cao et al. [59] found that calcium-rich mineral phases and C–S–H gel could immobilize Be and lead (Pb) through surface and interlayer adsorption as well as physical encapsulation, achieving immobilization rates exceeding 95% for both elements. Zheng et al. [76] noted that Tl^+^ can substitute for Na^+^ in the aluminosilicate structure, while Tl^3+^ is immobilized through the formation of Tl–O bonds or Tl(OH)_3_ precipitates. Additionally, Li et al. [77] employed microbially induced calcium carbonate precipitation to solidify LRR, achieving immobilization rates of 96.3% for Be and 83% for Tl. While immobilization effectively reduces the mobility of hazardous elements, it simultaneously encapsulates valuable elements within the matrix, hindering their subsequent separation and recovery.

### 3.3. Sintered Products

**Figure 9:**
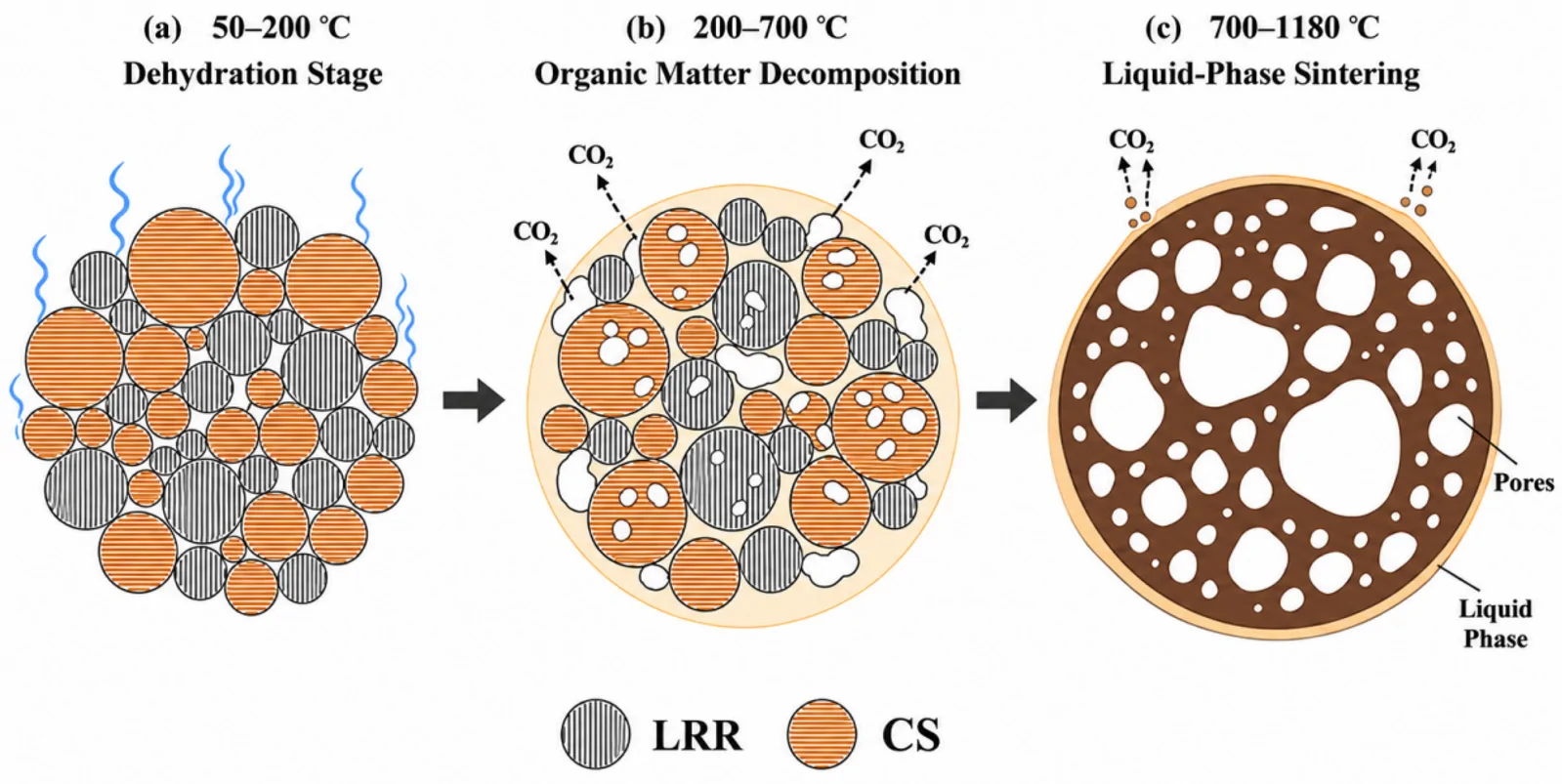
Process of preparing lightweight aggregate via the co-sintering of heavy metal-contaminated soil (CS) and LRR [78].

In sintered products, the Si-Al-rich phases of LRR mainly provide the ceramic framework, whereas alkali-bearing components can promote liquid-phase formation and facilitate sintering. High-temperature mineral-phase reconstruction and densification then convert LRR into products with adequate strength and stability. Gao et al. [78] co-sintered LRR with heavy-metal-contaminated soil to produce lightweight aggregates. The addition of LRR lowered the sintering temperature and enabled the synergistic treatment of the two solid wastes. The sintering process is illustrated in Figure 9. Li et al. [79] produced ceramsite by firing a mixture primarily composed of LRR and fly ash at 1240 °C, yielding aggregates with a bulk density of 0.46 g/cm^3^ and a water absorption of 0.96%. While this approach allows for high incorporation rates, it entails high energy consumption, and the unit price of the product is comparable to that of conventional building materials.

The stable crystalline phases and dense matrix formed during sintering can reduce metal leaching through incorporation into crystal lattices, solid-solution formation and physical encapsulation [78,79]. However, volatile elements such as S, F, and Tl may be released at high temperatures, causing secondary pollution [60]. Thus, attention must be paid to the capture and treatment of off-gases.

Overall, the sintering route is better suited for LRR with favorable fluxing or foaming properties and for regions possessing an existing infrastructure for ceramic or lightweight aggregate production. This method offers strong adaptability regarding raw material composition and allows for high incorporation rates of LRR. Furthermore, it can mitigate the leaching of harmful elements through densification and crystalline phase reconstruction. However, its economic and environmental viability depends heavily on sintering temperatures, product yields, and energy costs. Key bottlenecks hindering the industrialization of this route include high energy consumption associated with high temperatures, limited price advantages for the final products, and potential flue gas pollution caused by volatile components such as S, F, and Tl. Consequently, further research into selective fractionation and high-value utilization is warranted.

## 4. Selective Fractionation and Component-Oriented Valorization of LRR

In contrast to bulk utilization, selective fractionation aims to exploit differences in the occurrence and physicochemical properties of individual components, enabling their targeted recovery and subsequent product-oriented valorization. This strategy can be viewed as comprising three main stages. First, hazardous constituents such as Be, Tl, and F are removed or controlled to reduce environmental risks and avoid contamination of downstream products. Second, target components are selectively separated or recovered according to their modes of occurrence and separability, including Ca-S and Fe-bearing phases as well as residual valuable elements such as Li, Ta, Nb, Rb, and Cs, while the Si-Al-rich fraction is progressively purified. Finally, the separated fractions are directed toward suitable higher-value products according to their composition and quality requirements.

**Figure 10:**
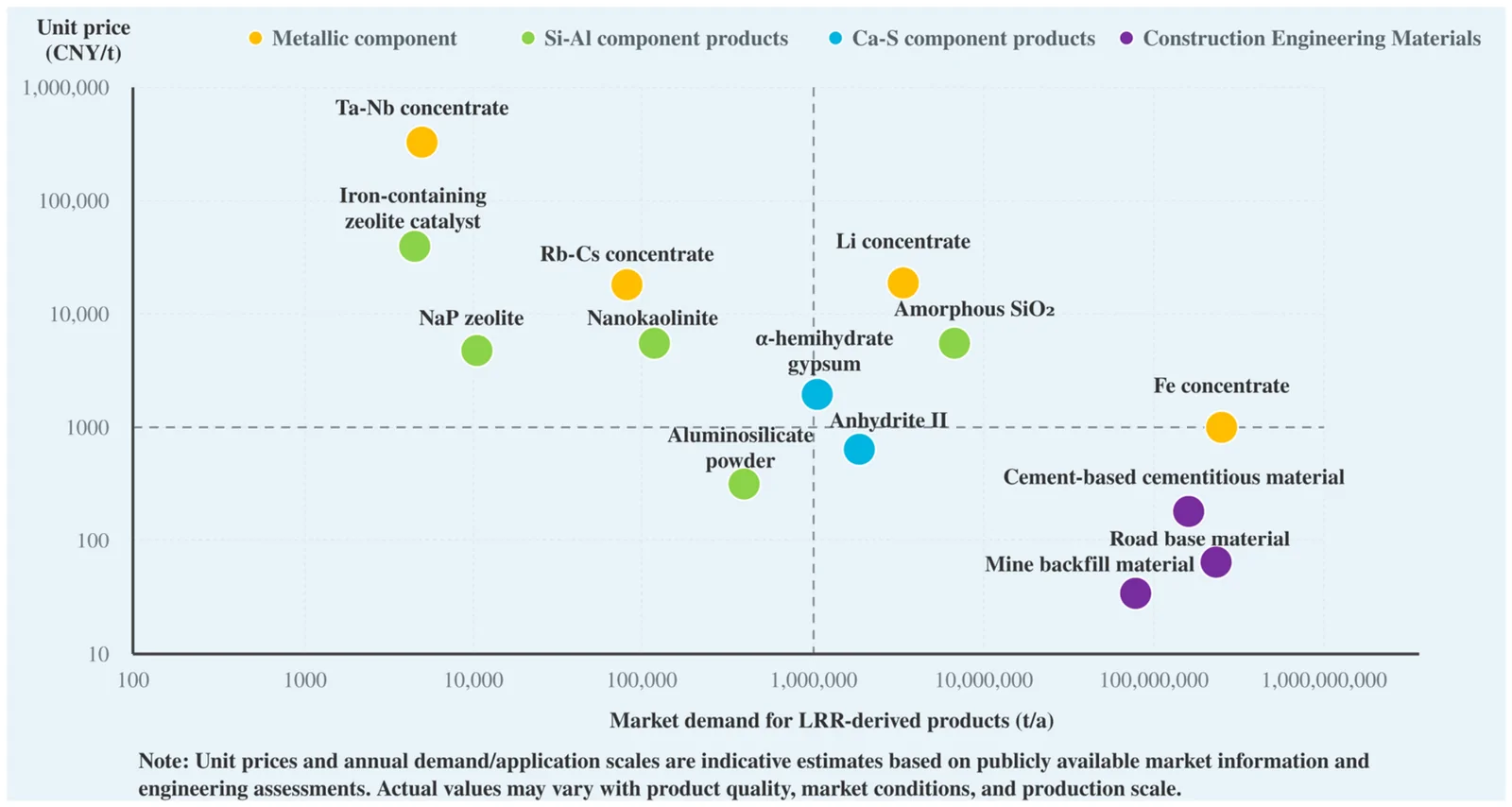
Market positioning of LRR-derived products by unit price and market demand.

As shown in Table 2, the metal content in LRR is relatively low. However, lepidolite-derived LRR contains high levels of rubidium (Rb) and cesium (Cs). The market prices and demand for products recovered from LRR are also analyzed at the end of this section (Figure 10).

### 4.1. Removal of Harmful Components

Trace hazardous elements in LRR—such as Be, Tl, and F—not only pose environmental risks but also compromise product stability. Therefore, they must be removed prior to selective fractionation. Unlike low-value immobilization and sequestration, removal involves the actual separation of these hazardous elements.

**Figure 11:**
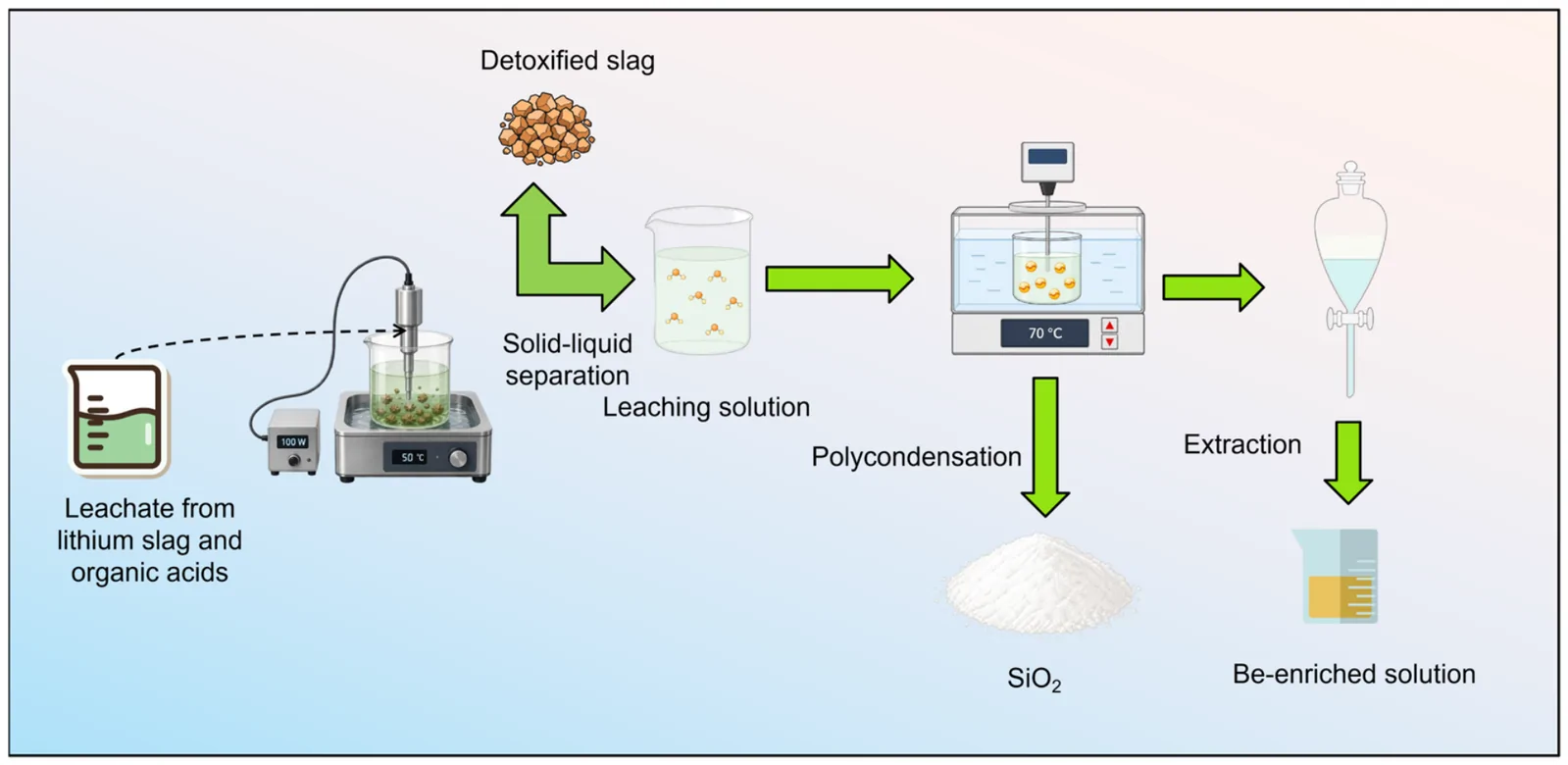
Schematic diagram of the experimental process for ultrasound-assisted leaching of Be using oxalic acid and citric acid [82].

Be occurs primarily in aluminosilicate and sulfate phases, whereas Tl is partly associated with iron–manganese oxides. Some of these Be-bearing and Tl-bearing phases are encapsulated within gypsum dihydrate (CaSO_4_·2H_2_O) [48,81,82]. F mainly exists as sparingly soluble CaF_2_, with some incorporated into the anorthite crystal lattice, alongside small amounts of water-soluble fluoride salts such as NaF and KF [46,47]. Based on these differing modes of occurrence, soluble F can first be removed via water washing, followed by the use of organic acids to dissolve the gypsum encapsulation phase and leach out unstable Be and Tl, with microbubbles or ultrasound employed to enhance particle de-agglomeration and mass transfer. Cheng et al. [48] achieved the selective removal of unstable Be and Tl using 1.25% oxalic acid. For Tl hosted in iron-manganese oxides, Yu et al. [81] introduced hydroxylamine hydrochloride to reduce and release the element, achieving a leaching rate of 91.7%. Subsequently, Yu et al. [82] utilized ultrasound-enhanced oxalic-citric acid leaching to achieve a Be leaching rate of 99.1% (the experimental process is illustrated in Figure 11). Liang et al. [47] demonstrated the feasibility of simultaneously removing F, Be, and Tl through microbubble-enhanced treatment, achieving removal rates of 71.79%, 50.38%, and 67.52%, respectively. Focusing specifically on soluble F, Yang et al. [83] reduced its content to below 10 mg/kg through water washing, calcium salt treatment, and ultrasonication. During the process, water washing removed soluble fluoride, and calcium salts immobilized the remaining fluoride as CaF_2_ rather than completely removing total F.

Overall, removing enriched elements such as F, Be, and Tl from LRR is a crucial prerequisite for producing high-purity or environmentally sensitive products. While water washing combined with organic acid leaching is effective for removing soluble fluorides as well as Be and Tl encapsulated in gypsum, addressing harmful elements hosted within iron-manganese oxides or fine-grained aggregates requires methods such as reductive leaching or enhanced mass transfer. However, removal of F, Be, and Tl from the solid phase transfers these elements to liquid streams that require subsequent treatment. Most reported removal efficiencies were obtained from batch experiments and do not establish stable continuous operation. Scale-up should evaluate feedstock variability, slurry mixing, residence-time distribution, solid–liquid separation, and equipment fouling. If washing or leaching solutions are recycled, the accumulation of dissolved salts and hazardous elements must be monitored, with appropriate solution treatment and controlled purge. Continuous trials should report removal efficiencies over time, element-by-element mass balances, reagent make-up, and effluent quality. The long-term leaching stability of the treated solids requires separate evaluation.

### 4.2. Selective Separation and Recovery of Target Components

After the removal of hazardous constituents, the following discussion of selective fractionation is organized according to the occurrence characteristics and downstream significance of the remaining components. A hierarchical framework is adopted: Ca-S and Fe-bearing components are first considered because their removal can improve the quality of the dominant Si-Al fraction, whereas residual valuable elements, including Li, Ta-Nb, and Rb-Cs, are subsequently discussed according to their respective modes of occurrence and separability. Depending on the target component and feed characteristics, physical or physicochemical methods, such as flotation, magnetic separation, and gravity separation, and chemical methods, including leaching and ion exchange, may be employed individually or in combination [84,85,86]. The final subsection then discusses representative integrated flowsheets in which compatible separation methods are selected and combined for the coordinated recovery of multiple components.

#### 4.2.1. Separation of Ca-S Components

**Figure 12:**
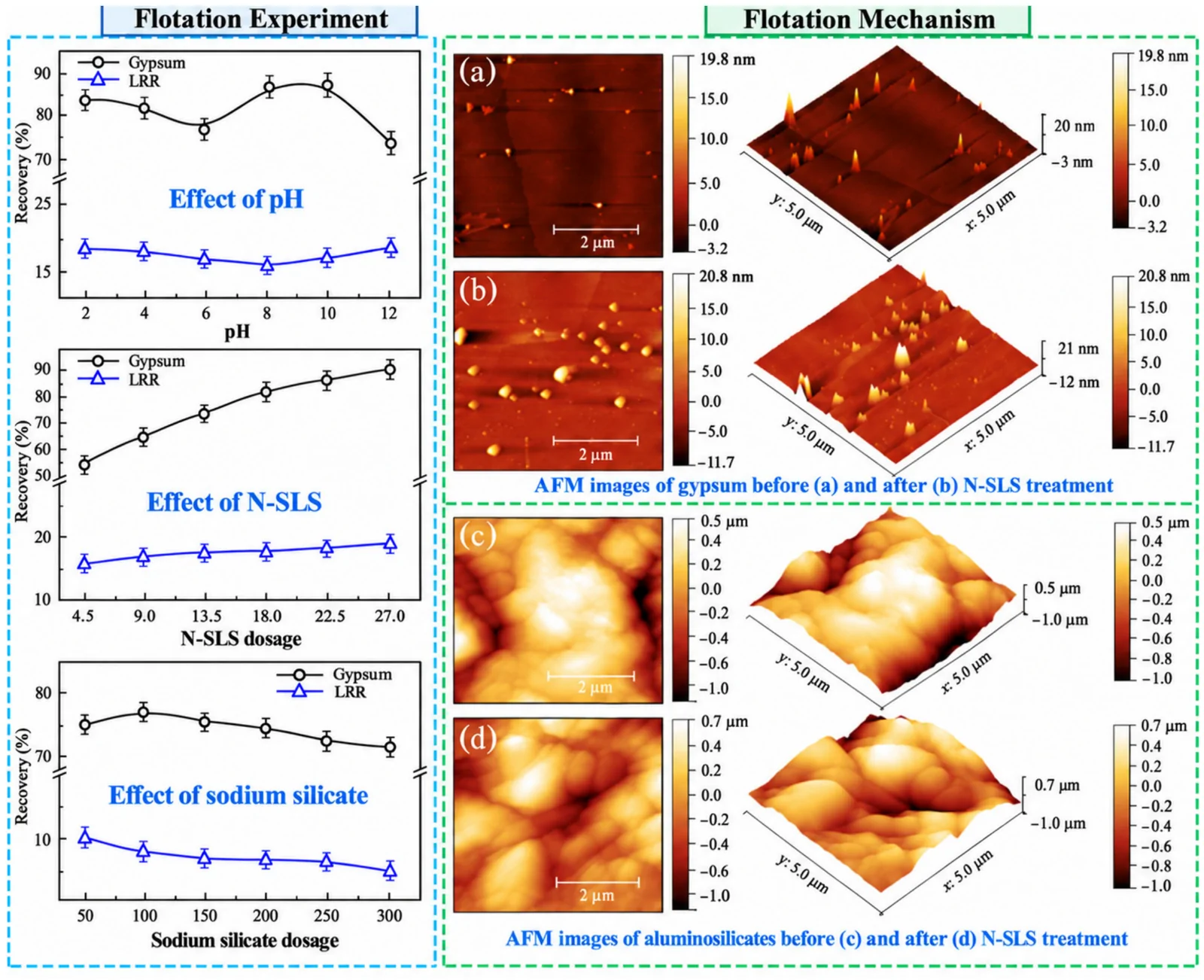
Flotation experiments and mechanism of collector N-SLS [89]: (**a**) Gypsum before N-SLS treatment; (**b**) Gypsum after N-SLS treatment; (**c**) Aluminosilicates before N-SLS treatment; (**d**) Aluminosilicates after N-SLS treatment.

The Ca-S components in LRR occur primarily as gypsum dihydrate. These components affect the hydration behavior and long-term durability of Si-Al-derived products in cementitious systems, thereby limiting the proportion of LRR that can be incorporated into construction materials [87,88]. Consequently, separating these Ca-S components is essential. Current separation methods rely predominantly on flotation, requiring both collectors and depressants. Collectors function through complexation between their carboxylate groups and calcium ions on the gypsum surface, rendering it hydrophobic and causing it to float, whereas depressants maintain the hydrophilicity of Si-Al minerals, keeping them in the pulp [88]. Guan et al. [89] developed an eco-friendly gypsum collector, sodium N-lauroyl sarcosinate (N-SLS) (shown in Figure 12). Optimal pH levels and N-SLS dosages significantly enhanced gypsum recovery while maintaining low flotation rates for the Si-Al residue, demonstrating excellent separation selectivity. AFM analysis indicated that the selective adsorption of N-SLS onto the gypsum surface was the primary mechanism driving this separation. Yi et al. [88] used sodium silicate (3000 g/t) as a depressant and a binary mixed collector (component mass ratio = 4:1, total dosage = 150 g/t) for the reverse flotation of gypsum, obtaining a gypsum concentrate with an SO_3_ grade of 42.56%. Building on this, a patented collector—a blend of C8–C20 fatty acid salts and dodecyl sulfonate—offered superior selectivity, achieving gypsum recovery rates of 95–99% and reducing the SO_3_ content in the slag to below 0.1% [90,91]. Alternatively, Ca-S components can be separated via dissolution. He et al. [87] employed a process involving hot dissolution in hydrochloric acid followed by cooling crystallization. By dissolving the material at 80 °C and subsequently recrystallizing it through cooling, they obtained gypsum with a purity of 99.79%. However, this method entails considerable acid consumption and requires the treatment of the resulting waste liquor. The reported flotation and dissolution results cannot be directly ranked by their numerical values because they emphasize different performance indicators. Flotation primarily targets gypsum recovery and concentrate grade, whereas dissolution-recrystallization can achieve high product purity but at the expense of greater acid consumption and subsequent waste-liquor treatment. In addition, Yan et al. [92] combined two-stage magnetic separation with acid leaching, reducing the sulfur and iron contents to 0.58% and 0.11%, respectively, thereby achieving the simultaneous removal of sulfur and iron. Beyond Ca-S removal, Fe-bearing impurities represent another important constraint on the quality of downstream Si-Al products and therefore require targeted separation.

#### 4.2.2. Separation of Fe Components

Iron-bearing impurities reduce the fired whiteness of ceramic products and limit the application of LRR in industries such as fiberglass manufacturing [86,92]. Consequently, iron removal is a crucial step for enhancing product quality. Integrated processing flows typically employ a two-stage magnetic separation approach—combining low-intensity and high-intensity magnetic separation—to first remove strongly magnetic iron minerals and subsequently concentrate weakly magnetic Ta and Nb minerals [16,86]. High-gradient magnetic separation can be utilized to further remove fine, weakly magnetic iron-bearing particles. In a study by Chen et al. [93], a single pass of magnetic separation—conducted with a background magnetic field strength of 1 T and a slurry flow velocity of 0.7 cm/s—reduced the Fe_2_O_3_ content in LRR from 0.96% to 0.34% and increased the fired whiteness from 18.8% to 45.66%, thereby meeting the requirements for ceramic raw materials. Removal of the major impurity phases improves the quality of the bulk Si-Al fraction. However, a second objective of selective fractionation is the recovery of residual valuable elements. These elements require different strategies because of their distinct modes of occurrence.

#### 4.2.3. Secondary Recovery of Li Components

The secondary recovery of lithium is constrained by its low residual content and the presence of impurities such as potassium and sodium. In particular, the co-leaching of these impurities increases acid consumption and interferes with subsequent separation and lithium precipitation [94]. Therefore, lithium recovery must balance leaching efficiency and selectivity against reagent consumption. Acid leaching is the primary recovery method. For example, Liu et al. [94] promoted H^+^/Li^+^ exchange and the dissolution of residual lithium using 2 mol/L hydrochloric acid at 40 °C, achieving a lithium leaching rate of 80.35%. For LRR that responds poorly to direct acid leaching, roasting activation can be employed to enhance lithium leachability. Yan et al. [95] modified the phase structure of lithium-bearing materials via roasting at 900 °C and utilized ultrasound-assisted leaching to achieve a lithium leaching rate of 82.47%. Although the two leaching efficiencies were obtained from different feed materials and operating conditions, their relatively close values (80.35% and 82.47%) indicate that the improvement achieved by roasting should be weighed against the additional energy input required at 900 °C. Roasting-assisted leaching is therefore more appropriate when refractory Li-bearing phases limit direct acid leaching, rather than as a universal pretreatment.

**Figure 13:**
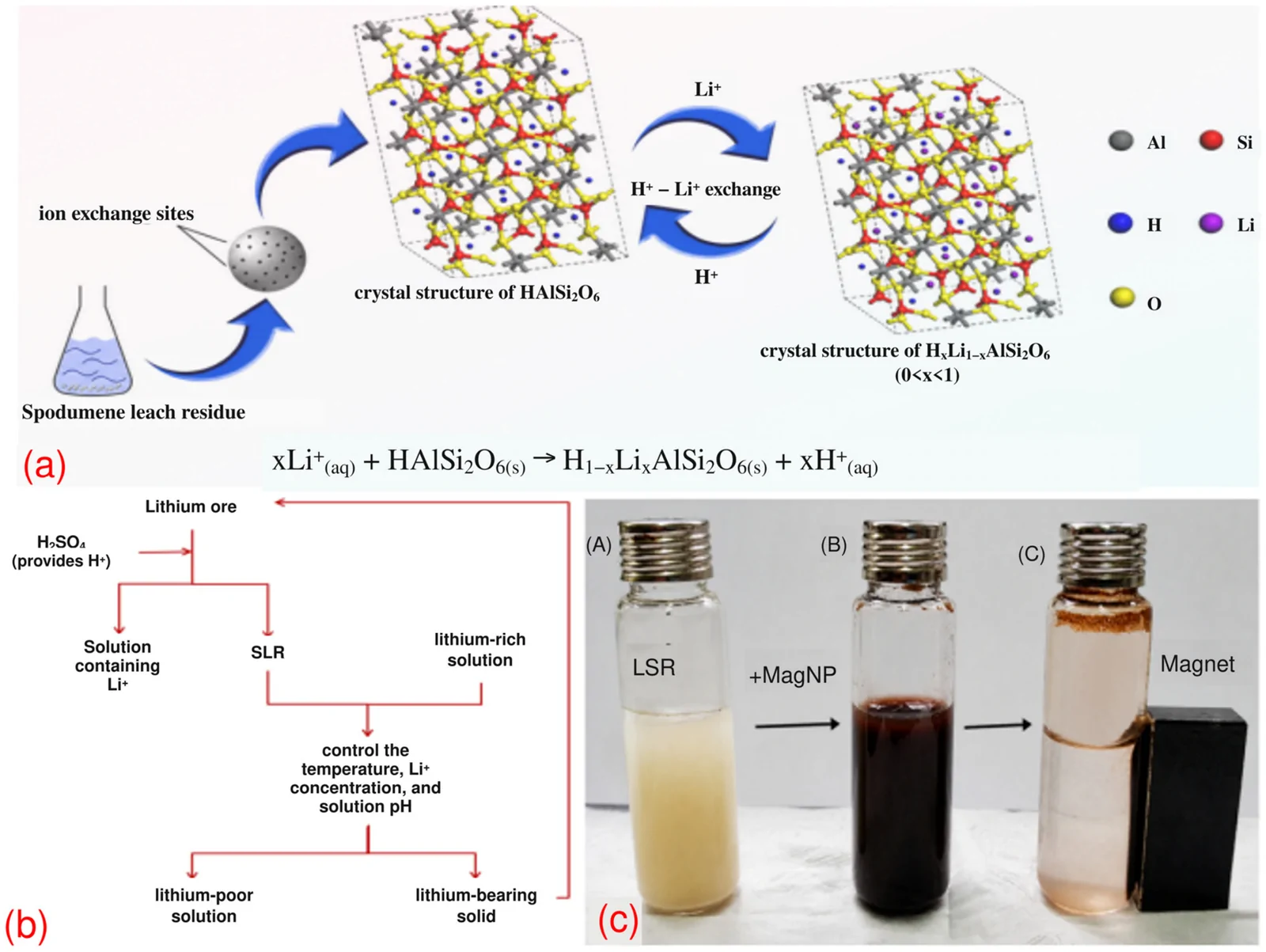
LRR as an adsorbent material and its cyclic regeneration mechanism: (**a**) Lithium enrichment via H^+^/Li^+^ lattice exchange [96]; (**b**) Ion exchange–acid leaching regeneration cycle [98]; (**c**) Magnetic nanoparticle-assisted separation of H-type LRR: (A) Acid-treated LRR suspension before nanoparticle addition; (B) Suspension after the addition of functionalized magnetic nanoparticles; (C) Magnetic separation of the nanoparticle-bound H-type ion-exchange material using an external magnet [99].

Acid-treated spodumene-derived LRR can also be used as a sorbent material to enrich lithium from lithium-bearing solutions. Han et al. [96] discovered that acid-treated LRR could selectively adsorb lithium from brine via an H^+^/Li^+^ exchange mechanism (as shown in Figure 13a). For such slag-derived adsorbents, Asif et al. [97] reported an adsorption capacity of 14.25 mg/g. Building on this approach, Wang et al. [98] enhanced the H^+^/Li^+^ exchange process by adjusting the solution alkalinity, thereby increasing the Li_2_O content in the residue to 8.44%. As illustrated in Figure 13b, after Li^+^ desorption by acid elution, the lithium-enriched solid phase could be regenerated as an H-type ion-exchange material, establishing an “ion exchange–acid leaching” regeneration cycle. Notably, the residue remaining after acid leaching is not pure H-β-spodumene but contains quartz, gypsum, and other impurities. Quartarolli et al. [99] utilized functionalized magnetic nanoparticles to bind selectively to protonated β-spodumene, achieving the separation and purification of the H-type exchange material through an external magnetic field (as shown in Figure 13c). Although this method expands the utilization pathways for LRR, there remains room for improvement regarding adsorption capacity and cycling stability. Furthermore, studies on lithium-bearing ores and mine tailings suggest that physical beneficiation may be applicable when residual lithium-bearing minerals are sufficiently liberated [100,101,102], whereas LRR generated from molten salt electrolysis can be recovered through vacuum distillation [103]. Therefore, the appropriate recovery method should be selected based on the form of the residual lithium and the characteristics of the raw material. Unlike residual Li, which is mainly recovered through chemical activation, leaching, or ion-exchange-related processes, Ta and Nb are commonly associated with discrete mineral phases and can therefore be enriched by exploiting their magnetic and density differences.

#### 4.2.4. Separation of Ta-Nb Components

**Figure 14:**
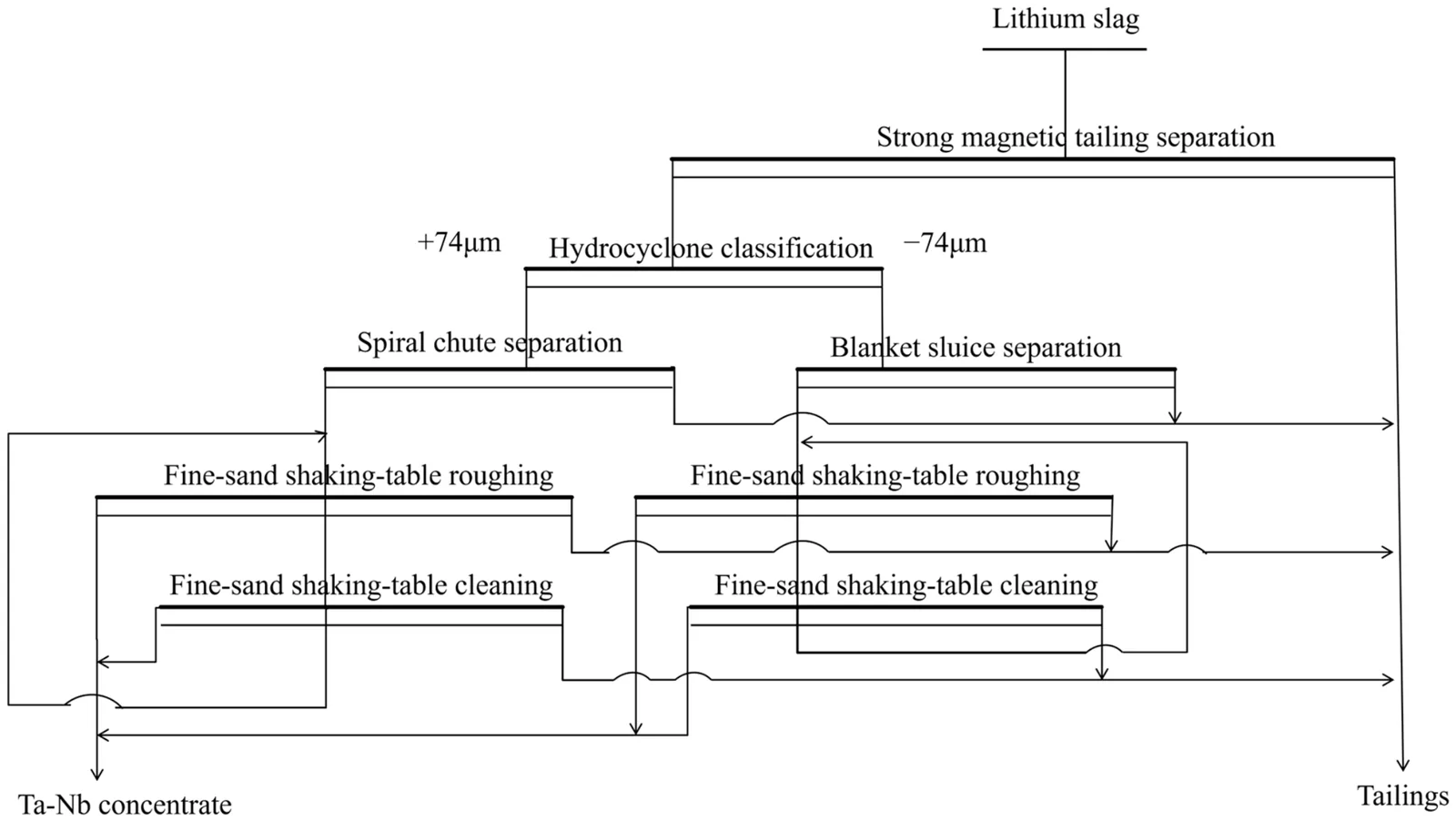
Flowsheet for the magnetic separation and classification-gravity separation of Ta and Nb components from LRR [80].

Ta-Nb minerals are often encapsulated in a glass phase or intergrown with aluminosilicate minerals in a finely dispersed state, making liberation and separation challenging [80]. Deng et al. [80] determined that Ta and Nb in LRR occur primarily as weakly magnetic, high-density tantalite–columbite. Based on this finding, they employed a closed-circuit process comprising magnetic separation and gravity separation. High-intensity magnetic separation was first used for slag rejection, followed by gravity separation on size-specific shaking tables (Figure 14). The process produced a concentrate containing 20.87% Ta_2_O_5_ and 16.24% Nb_2_O_5_, with recoveries of approximately 52–54%. However, multistage gravity separation often results in the loss of fine particles. Beyond studies conducted directly on LRR, research on other Ta-Nb-bearing feedstocks provides useful methodological insights for improving mineral liberation and fine-particle recovery. Cheng et al. [104] investigated magnetic-fluid-assisted separation of Ta-Nb minerals associated with spodumene ore. They used a manganese chloride-based magnetic fluid as the separation medium and adjusted its magnetic susceptibility to enhance the magnetic contrast between minerals, thereby achieving better fine-particle recovery than gravity separation. Hassan et al. [105] achieved Ta-Nb recoveries exceeding 94% from fine-grained Abu Dabbab ore using Falcon centrifugal separation combined with magnetic separation. Additionally, Allain et al. [106] employed sequential acid-alkali leaching to selectively dissolve the amorphous matrix of tin slag, thereby enriching the Ta-Nb in the residue. Combined beneficiation processes for Ta-Nb-bearing lepidolite ores also provide useful references [107,108]. Overall, the physical enrichment of Ta and Nb from LRR relies primarily on magnetic and gravity separation, with adequate liberation and efficient recovery of fine-grained Ta and Nb remaining the critical factors.

#### 4.2.5. Separation of Rb-Cs Components

In contrast to the physical enrichment of Ta-Nb-bearing minerals, Rb and Cs occur mainly through isomorphous substitution for K in lepidolite and related aluminosilicates. Their lattice-bound occurrence makes direct physical beneficiation ineffective; therefore, hydrometallurgical leaching is generally preferred. However, the high Si and Al contents of LRR can hinder the selective extraction of Rb and Cs. Zhong et al. [14] utilized sulfuric acid to disrupt the lepidolite structure and release Rb from the mineral lattice, achieving a Rb leaching rate of 86.26% at 85 °C. While the process is simple, it entails high acid consumption. In a related study using lepidolite rather than LRR, Zhou et al. [109] employed alkaline pressure leaching with calcium hydroxide to promote lepidolite decomposition, immobilizing Si and Al in the residue while simultaneously transferring Li, Rb, and Cs into the leachate. Subsequent solvent extraction from the leachate yielded recovery rates of 92.7%, 94.3%, and 94.5% for Li, Rb, and Cs, respectively. The higher reported Rb-Cs recoveries of the alkaline pressure route should be interpreted in the context of its more intensive process configuration. Sulfuric-acid leaching mainly relies on direct disruption of the aluminosilicate lattice at relatively mild temperatures, whereas alkaline pressure treatment combines pressure-assisted mineral decomposition with subsequent solvent extraction, thereby promoting higher overall recovery but requiring more demanding operating conditions. Yu et al. [15] applied an ultrasonic-assisted polycondensation–coprecipitation method to remove silicic acid via gelation while simultaneously releasing encapsulated Rb and Cs. Overall, efficient Rb-Cs recovery requires both disruption of the host aluminosilicate structure and effective control of Si-related interference during leaching.

#### 4.2.6. Integrated Separation of Multiple Components

**Figure 15:**
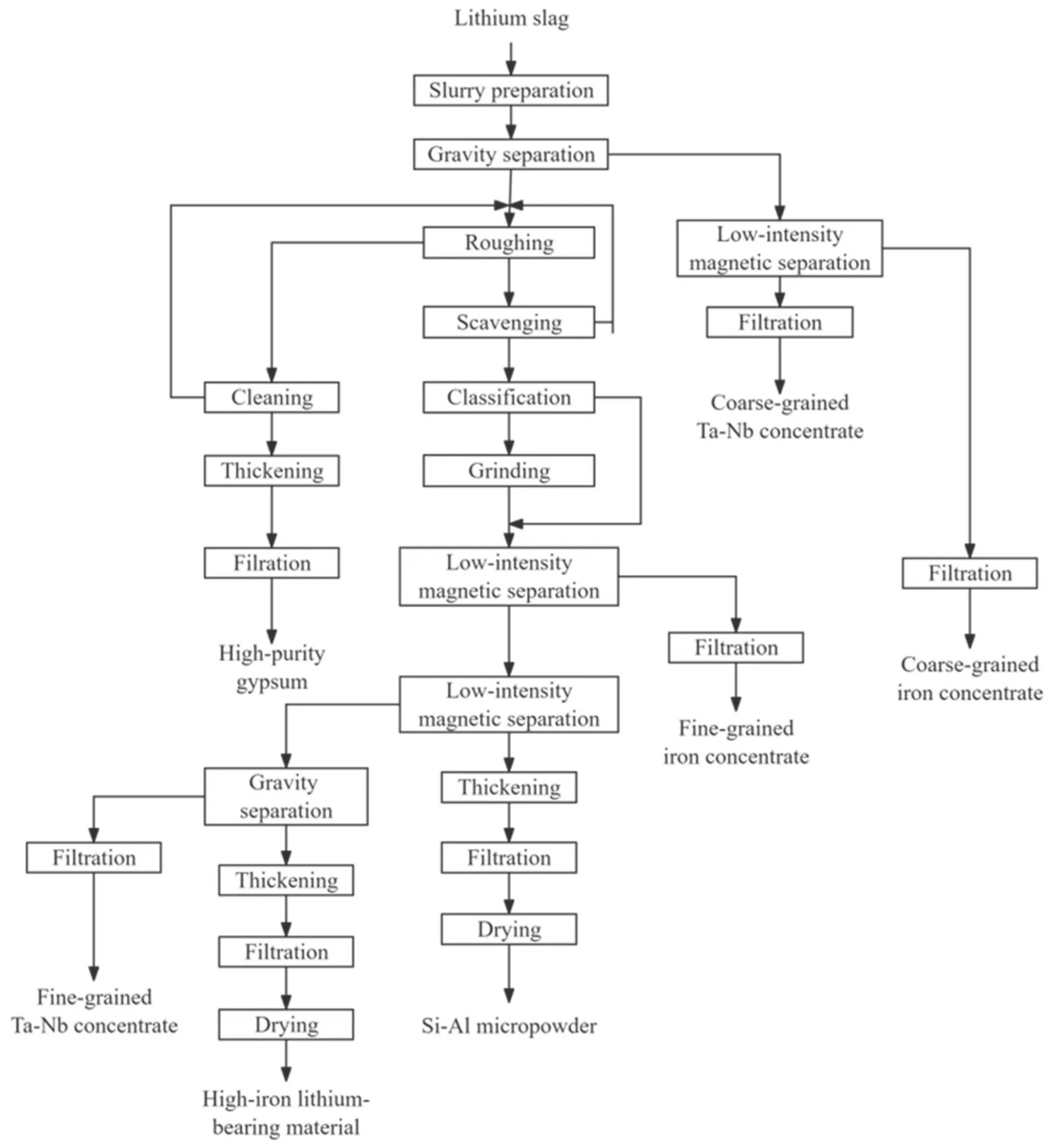
Flowchart of an integrated separation process for multiple components of LRR [110,111,112].

The preceding subsections summarize the principal separation strategies available for different target components in LRR, based on their distinct modes of occurrence and physicochemical properties. In practical valorization, these techniques are not necessarily applied as independent or sequential unit operations; rather, appropriate physical, physicochemical, and chemical methods are selected and combined according to the composition and mineralogical characteristics of the feed. Integrated flowsheets therefore provide a more realistic route for the coordinated recovery of multiple components from LRR. Zhang et al. developed an integrated separation process for multiple components of LRR [86,110]. Following grinding and liberation, low-intensity magnetic separation yields iron concentrate, while high-intensity magnetic separation—followed by gravity separation and refining—enriches Ta and Nb. Subsequent flotation removes sulfur to produce gypsum, and alkaline conversion of the slag yields aluminosilicate powder. This integrated process generates four types of products, as illustrated in Figure 15. Similar processes have also been described in several patents [110,111,112]. Notably, Tianqi Lithium and its affiliated company have developed patented integrated LRR-processing routes for the coordinated recovery of Si-Al-rich products, gypsum, Ta-Nb concentrates, and Fe-bearing fractions [86,110,111,112]. And the company’s industrial application of the LRR integrated processing technology provides a prime example of selective fractionation and multi-component recovery in practice. Overall, the integrated separation process combines magnetic and gravity separation, flotation, and alkaline conversion to enable the coordinated recovery of iron concentrate, Ta-Nb concentrate, gypsum, and aluminosilicate powder from a single LRR feedstock, thereby integrating rare-metal recovery with the utilization of bulk components. Compared to single-component recovery, this process allows for shared grinding and separation operations, reduces residue generation, and distributes processing costs across the revenues of multiple products, making it a representative system solution for the selective fractionation of LRR. However, broader application requires adaptation to feedstock variability and improved recovery of fine-grained Ta-Nb minerals. Effective coordination of unit operations and consistent product quality are also essential. Further work should therefore focus on long-term operational performance, element-specific mass balances, and comprehensive techno-economic evaluation under industrial conditions.

### 4.3. Product-Oriented Valorization of Separated Components

Following selective separation and purification, the recovered fractions must be matched with appropriate downstream products according to their composition, purity, and functional properties. Accordingly, this section focuses on the product-oriented valorization of the separated Si-Al, Ca-S, and other valuable components.

#### 4.3.1. High-Value Utilization of Si-Al Components

**Figure 16:**
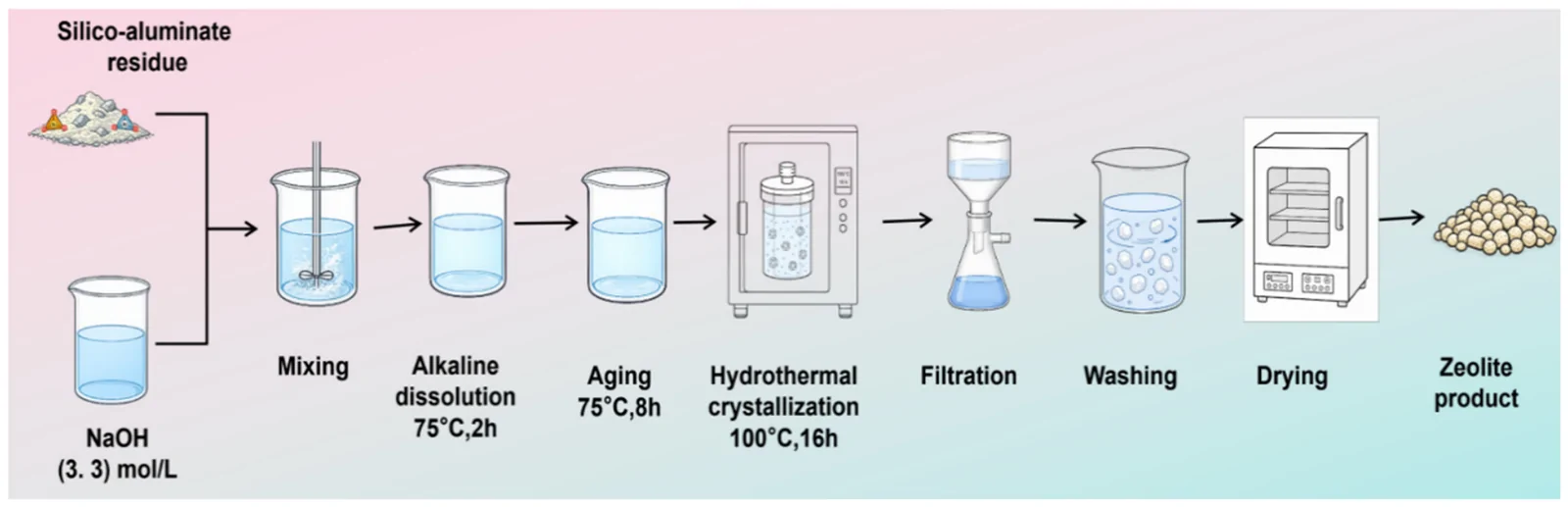
Process flow for the hydrothermal synthesis of zeolite from Si-Al residue [117].

The Si-Al components constitute the largest proportion of LRR, typically accounting for more than 70% of its composition. Consequently, achieving the valorization of these components addresses the primary obstacle to the overall valorization of LRR. There are two main pathways for the high-value utilization of Si-Al components in LRR: whole-residue conversion and separation and purification. Whole-residue conversion encompasses the production of porous ceramics, zeolites, and aluminosilicate powder. Xiong et al. [113] produced porous ceramics with a strength of 5.70 MPa using added fluxing agents, although the flux diluted the LRR content. Conversely, Wang et al. [114] utilized the alkali metal components within the LRR to promote sintering and foaming, achieving a slag content of 90% and reducing the sintering temperature to 1225 °C, with a production cost of approximately 87 CNY/m^2^—about 21% lower than traditional processes. Notably, lepidolite-derived LRR is prone to foaming due to its high alkali content, whereas spodumene-derived LRR is limited by its high quartz content and low alkali content [37]. Market quotations indicate that foamed ceramic insulation boards sell for approximately 150–350 CNY/m^2^. This price is substantially higher than the estimated production cost, suggesting that this production route may be economically viable. Jin et al. [115] synthesized iron-containing zeolite catalysts via alkali treatment and hydrothermal crystallization, achieving a tetracycline degradation rate approximately three times that of the raw slag. Alkali fusion activation also allows for the modulation of the zeolite’s phase structure [116]. As shown in Figure 16, Ibsaine et al. [117] converted aluminosilicate residues from lithium processing in Québec, Canada, into NaP-type zeolite through alkali dissolution, aging, and hydrothermal crystallization. Its estimated production cost was approximately 0.62 USD/kg, compared with a selling price of around 1.00 USD/kg, indicating potential economic viability. In comparison, the preparation of aluminosilicate powder is relatively straightforward and closer to industrial deployment. These powders can be produced by subjecting flotation desulfurization slag to alkali conversion [86,118]. Shui [119] patented a process for converting LRR into aluminosilicate powder through desulfurization and iron removal. The resulting powder can serve as a raw material for fiberglass production and partially replace natural pyrophyllite. Its estimated production cost is approximately 180–190 CNY/t, compared with a contemporaneous market price of approximately 620 CNY/t for natural pyrophyllite micropowder, suggesting a potential cost advantage.

Separation and purification processes are primarily employed to produce high-value products such as silica, kaolinite, and xonotlite. Zhao et al. [120] produced amorphous SiO_2_ with a purity of 87.85% by using acid pretreatment to leach out impurities and disrupt the silicate structure, followed by alkaline leaching in 5 mol/L NaOH at 85 °C. Similarly, Wang et al. [52] prepared nanokaolinite by hydrothermally decomposing LRR with NaOH at 220 °C and synthesized xonotlite by reacting the desilication solution with CaO. Combined processes involving magnetic separation, gravity separation, and hydrothermal treatment can also achieve the fractionation and recovery of different components [121]. Although batch experiments demonstrate the feasibility of producing Si-Al-derived products, industrial scale-up requires control of feed composition, slurry rheology, mixing and heat transfer, crystallization or precipitation, filtration, and drying. Sustained operation should be assessed through throughput, qualified-product yield, purity, crystal phase, and catalytic performance where applicable. These indicators, together with reagent consumption and the quality of recycled process liquors, need to remain stable over time.

#### 4.3.2. High-Value Utilization of Ca-S Components

**Figure 17:**
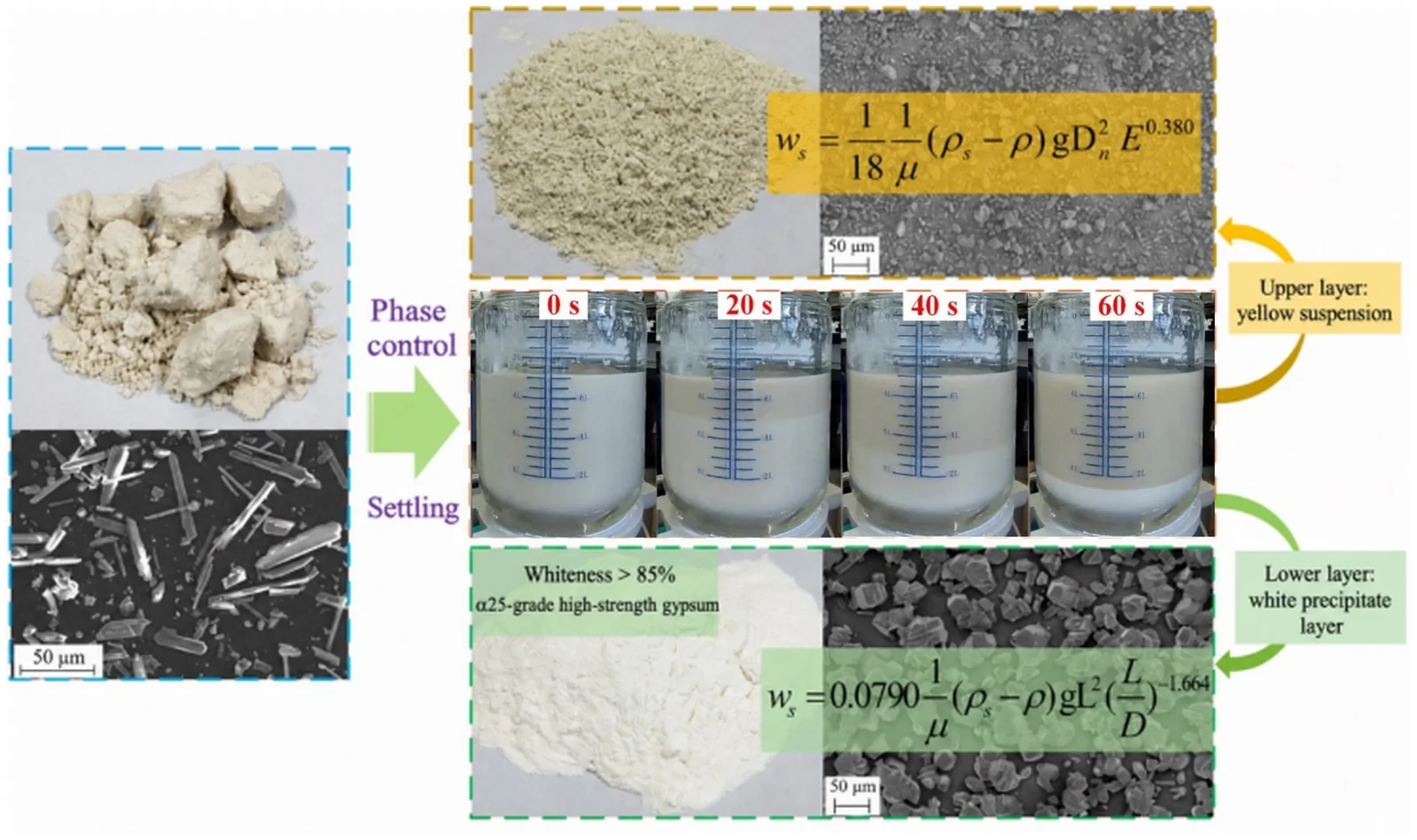
Process for preparing high-whiteness, high-strength α-hemihydrate gypsum from the gypsum-rich component recovered from LRR [124].

High-strength α-hemihydrate gypsum and anhydrite II can be prepared from Ca-S components through impurity removal via phase transformation and crystal morphology control [122,123]. Guan et al. converted the gypsum-rich component recovered from LRR into high-strength α-hemihydrate gypsum under atmospheric pressure. During the transformation of gypsum dihydrate to α-hemihydrate gypsum, encapsulated fine-grained aluminosilicate impurities were released. Simultaneously, sodium succinate regulated the crystal morphology, promoting the formation of relatively large, rapidly settling, near-spherical α-hemihydrate gypsum particles that could be separated from the suspended fine aluminosilicate impurities, ultimately yielding α25-grade high-strength gypsum with a whiteness of 86.3% (Figure 17) [89,124]. This method is also applicable to the removal of lattice-bound phosphorus from phosphogypsum and lattice-bound arsenic from arsenic-bearing gypsum, relying on the principle of impurity separation through phase transformation and reconstruction [125,126,127,128]. Furthermore, the purified gypsum can be converted into anhydrite II via high-temperature calcination or high-pressure hydrothermal treatment, serving as an inorganic filler for industries such as papermaking and rubber manufacturing [129]. However, for LRR-derived Ca-S products, continuous phase transformation and impurity separation must also be validated at stable throughput, while monitoring crystal morphology, filterability, product whiteness and strength, and impurity accumulation in recycled process media. These operational requirements may also affect production costs. Accordingly, Table 3 summarizes the production costs and market selling prices of products derived from Si-Al components and Ca-S components. Production costs were estimated based on process parameters and the prices of raw materials, reagents, and energy reported in the literature, while selling prices reflect market quotations. Actual prices are influenced by factors such as product quality, production scale, and market supply and demand. Therefore, the data presented in the table are intended solely for preliminary economic comparison and do not represent actual production costs or final transaction prices.

Under the assumed conditions, the estimated production cost of amorphous SiO_2_ substantially exceeds its indicative market price, mainly because the reported process involves acid pretreatment, concentrated alkaline leaching, precipitation, and subsequent thermal treatment. This large cost-price gap highlights the need for reagent recovery, process optimization, and comprehensive techno-economic assessment before industrial implementation. In contrast, iron-containing zeolite catalysts show a considerably higher indicative market value than the estimated production cost. However, this apparent economic advantage should be interpreted cautiously, as fluctuations in downstream demand and reagent losses during continuous operation and recycling may substantially affect the actual process economics.

#### 4.3.3. Product Positioning of Other Components

After separation, enrichment, and purification, components containing Ta-Nb, iron, lithium, and Rb–Cs can be converted into marketable concentrates or salts, as appropriate. Ta, Nb, Rb, and Cs are high-value strategic metals used in sectors such as electronics, aerospace, and precision instrumentation. Meanwhile, high-quality iron concentrate can serve as a raw material for iron and steel production, whereas recovered lithium salts can be reintegrated into the lithium industry chain. Consequently, combining the recovery of trace valuable components with the utilization of bulk Si-Al and Ca-S components balances economic benefits and overall valorization efficiency, collectively forming a system for the high-value utilization of LRR.

In general, the selection of high-value products should be based on the purity, mineral composition, and performance characteristics of the separated components, rather than solely on unit price. Si-Al components of moderate purity are better suited for producing aluminosilicate powder or porous ceramics, whereas the production of zeolites, amorphous SiO_2_, and nano-kaolinite demands higher raw material purity and stricter process control. Purified Ca-S components are suitable for manufacturing high-strength gypsum, while rare metal components are best recovered as by-products within an integrated process. Currently, the production technologies and industrial applicability for porous ceramics and gypsum products are relatively mature. In contrast, the production processes for zeolites, amorphous SiO_2_, and nano-kaolinite are more complex—requiring high raw material purity, precise reaction conditions, and consistent product quality—and the feasibility of their industrial production requires further validation (Table 4).

## 5. Conclusions

This paper provides a comprehensive review of the sources and properties of LRR, as well as technologies for low-value disposal, activation and modification, selective fractionation, and the preparation of high-value-added products. The main conclusions and future outlook are summarized below:

(1) LRR can be used to produce roadbed and mine-backfill materials, cement-based materials, geopolymers, and sintered products. This bulk-utilization strategy offers high consumption capacity, strong process adaptability, and a well-established industrial foundation.

(2) Controlling harmful elements is a necessary prerequisite for producing high-value-added products from lithium refinery residues. Methods such as selective leaching using organic acids show promise for removing harmful elements like Be and Tl. However, industrial application still requires careful consideration of reagent consumption and processing costs. Particular attention should also be given to the stability of F, Be, and Tl removal during continuous operation, their accumulation in recycled process liquors, and the long-term leaching stability of the treated residues.

(3) Among high-value applications, porous ceramics exhibit relatively high technological maturity and industrial compatibility. In contrast, the production of materials such as zeolites, amorphous silica, and nanokaolinite involves more complex processes and imposes stricter requirements on raw material purity, reaction conditions, and separation equipment. Consequently, the industrial feasibility of these products remains to be validated through sustained pilot-scale operation, with particular attention to throughput, product yield, and consistent quality.

(4) From an economic perspective, some products derived from the Si-Al and Ca-S components show potential profitability, whereas the estimated costs of amorphous SiO_2_ currently exceed their indicative market prices. Further process optimization, reagent recycling, product quality improvement, and stable downstream markets are therefore essential. Efficient and low-cost selective enrichment technologies are also required to recover low-grade and finely disseminated Rb, Cs, Ta, and Nb.

(5) Selective fractionation is essential for transforming LRR from low-value utilization toward higher-value resource recovery. Based on the occurrence and physicochemical characteristics of different components, leaching, flotation, magnetic separation, and gravity separation can be selectively combined to recover Si-Al, Ca-S, and Fe-bearing fractions, as well as valuable elements such as Li, Rb, Cs, Ta, and Nb. However, further development is still limited by the large variability of LRR feedstocks, the difficulty in recovering low-grade and fine-grained valuable components, the insufficient coordination among individual separation steps, and the lack of long-term continuous operation and product quality validation. Future research should focus on improving the characterization of LRR, enhancing the recovery of fine-grained valuable components, optimizing integrated separation processes, and strengthening pilot-scale and economic evaluations to support industrial application.

## Figures and Tables

**Table 2:** Chemical composition and content of rare metals in LRR.

Types of LRR	Origin	Li_2_O (wt%)	Rb_2_O (wt%)	Cs_2_O (wt%)	Ta_2_O_5 _ (wt%)	Nb_2_O_5 _ (wt%)	Be (μg/g)	Tl (μg/g)
**Spodumene-derived LRR**	Australia [7]	0.09	0.18	0.11	-	-	-	-
	Sichuan [80]	-	-	-	0.0077	0.0062	-	-
**Lepidolite-derived LRR**	Jiangxi [7]	0.24	1.56	0.21	-	-	-	-
	Jiangxi [47]	0.10	-	-	-	-	19.97	2.74

Note: “-” indicates not reported in the original text.

**Table 3:** Estimated production costs and indicative market prices of products derived from LRR.

LRR Components	Product	Estimated Production Cost	Indicative Market Price
**Si-Al components**	Aluminosilicate powder	350 CNY/t	600 CNY/t
**Si-Al components**	Amorphous SiO_2_	34,000 CNY/t	8000 CNY/t
**Si-Al components**	Nano-kaolinite	7000 CNY/t	8000 CNY/t
**Si-Al components**	Zeolite NaP	4400 CNY/t	7200 CNY/t
**Si-Al components**	Iron-containing zeolite catalysts	10,900 CNY/t	80,000 CNY/t
**Si-Al components**	Xonotlite	5500 CNY/t	8750 CNY/t
**Si-Al components**	Porous ceramics	87 CNY/m^2^	350 CNY/m^2^
**Ca-S components**	α-hemihydrate gypsum	800 CNY/t	1300 CNY/t
**Ca-S components**	Anhydrite II	400 CNY/t	900 CNY/t

**Table 4:** Comparison of major LRR utilization and selective-fractionation routes.

Main Route	Advantages	Limitations	Environmental Aspects	Industrial Applicability
Direct incorporation	Low cost, high residue uptake	Low added value, dosage limits	Reduces stockpiling, but hazardous-element leaching remains a concern.	Field demonstration,
Activated cementitious utilization	Higher reactivity, clinker substitution	High activation costs, durability concerns	May reduce clinker-related CO_2_ emissions, but grinding or calcination requires energy.	Laboratory studies, compatible with cement infrastructure
Sintered products	High residue uptake, improved stability	High energy demand, limited price advantage	Can reduce metal leaching, but may release volatile pollutants.	Laboratory-scale testing
Ca–S and Fe separation	Higher Si–Al purity, by-product recovery	Feed-dependent separation, high acid consumption in dissolution-based purification	Reduces residue disposal, but acid-based routes may generate wastewater.	Laboratory studies and patented routes
Secondary Li, Ta–Nb, and Rb–Cs recovery	Valuable-metal recovery, increased resource value	Low metal contents, costly recovery	Conserves valuable metals, but chemical leaching may generate waste liquor.	Predominantly laboratory-scale
Integrated multi-component fractionation	Recovery of multiple fractions, less final residue	Complex process integration, unstable product quality	May reduce final residues, but grinding consumes energy and wet processing may generate wastewater.	Industrial-scale application

## Data Availability

No new data were created or analyzed in this study. Data sharing is not applicable to this article.
